# The role of cryptocurrency in the dynamics of blockchain-based social networks: The case of Steemit

**DOI:** 10.1371/journal.pone.0267612

**Published:** 2022-06-16

**Authors:** Cheick Tidiane Ba, Matteo Zignani, Sabrina Gaito

**Affiliations:** CONNETS Lab, Computer Science Department, Università degli Studi di Milano, Milan, Italy; University of Pisa, ITALY

## Abstract

A shift of paradigm is running over online social platforms: the over-centralization of these platforms is leaving room for decentralized solutions based on blockchain technologies, such as blockchain-based online social networks—BOSNs. Among the many unknown aspects of these techno-social systems, the objective of this study is to propose an analytical framework to assess the impact of the cryptocurrencies linked to a BOSN platform on the evolution of its social network and on the behavior of their users, in terms of production of content and/or its promotion through a voting and rewarding system. The framework has been applied to Steemit, one of the most widespread BOSNs, from which we collected three-year-long high-resolution data on its evolution along with the price of its main cryptocurrencies. On users’ activities extracted from these longitudinal data, we applied a time-series correlation analysis and a correlation analysis between the action allocation strategies and the obtained rewards, in the case of most central accounts. The analysis has highlighted pieces of evidence of the influence of the cryptocurrency price on users’ actions, particularly on actions that shape the structure of the social networks. Second, we also found highly rewarded users prefer actions related to the promotion of content rather than the creation of high-quality content, exploiting the reward distribution mechanisms implemented by the platform. These findings highlight that the shift of paradigm towards blockchain and cryptocurrency technologies might strengthen the influence of financial and economic factors rather than relational/social aspects on the evolution of these new complex techno-social systems.

## Introduction

We are currently witnessing a dramatic moment of crisis and deep renewal of the social media landscape induced by two opposite forces. On the one hand, these platforms are increasingly playing a fundamental role in many aspects of the life of human beings, especially in the new generations who continuously ask for new services. On the other hand, there is a growing awareness that the traditional model of centralized social networks is no longer sustainable and poses crucial challenges that require adequate and rapid solutions to the well-known issues of privacy, content quality, censorship, and data ownership and monetization.

Among the various possible solutions, one of the most promising are blockchain-based online social networks (BOSN), which put themselves forward as social platforms able to overcome all current issues of centralized social networks. Actually, three specific aspects, common to most of the current BOSNs, are: a decentralization based on blockchain technologies that mitigate data privacy and censorship problems, typical of centralized platforms [1] (a few blockchains online social networks combine blockchain decentralization and tokenization to replace sensitive and/or personal data or have introduced self custody wallets to keep data private; nevertheless, the immutability and transparency of blockchains still pose data privacy issues since transaction history of digital wallets can be completely reconstructed); a token system based on proprietary cryptocurrency used for fostering high-quality content; and a rewarding system for distributing the wealth of the platform giving data monetization back to users and encouraging good practices.

Despite having been around for a few years, we are very far from having fully understood to what extent BOSN paradigm solves the issues of traditional architectures and what are, if exist, the other problems they potentially introduce.

The true pivot of BOSN is the introduction of a cryptocurrency that shifts the paradigm of online social network from being purely social to economic-social: in the traditional approach, users are engaged with social interactions, while economic ones are prerogative of platform ownership; while in BOSN users are got dragged into social-economic actions. Thus, the way to understand the BOSN in-depth passes through the investigation of the relations between the economic and social actions carried out by users and how both relate to the value of the cryptocurrency.

To shed a light on this complex network of intertwined layers, we adopt a data-driven approach, by analyzing Steemit, one of the first and most successful BOSNs. By gathering data from the underlying blockchain Steem, we have collected a large longitudinal dataset that contains the main social and financial activities of Steemit users spanning more than three years, along with data external to the Steemit platform: longitudinal data of STEEM value in the cryptocurrency market. From these data we were able to reconstruct the high-resolution evolution of the system to address the main goal of our study: the interplay between users’ social and financial activities, resulting in social and economic networks, and the currency price; with a specific focus on the possible effects of the currency price on the network structure. As for this latter aspect, our analysis based on time series correlation has pointed out a possible influence of the platform cryptocurrency on the evolution of the Steemit social network, i.e. “follow” or link creation actions have been partly driven by the trend of the cryptocurrency. Higher prices have attracted more users and shifted the mechanisms and the strategies ruling link creation. Strategies and action allocation, especially for the most central nodes, are a further focus of our study. In particular, we highlighted which actions central nodes have mainly chosen to gain the highest cumulative rewards. Here, we observe that central nodes exploit both their high rank in the voting system and the mechanism of the rewarding system to get rewards, i.e. they tend to prefer voting operations to actions for producing content (posting and commenting).

The above findings suggest that the transformation of the actual online social platforms—which in the last years have shaped and are still changing our society—into new paradigms supported by blockchain technologies ask for new perspectives for the study of their evolution. Indeed, economic and financial aspects might play a more decisive role in how people behave in these new platforms, enough to question the relational aspects, typical of the main online social networks.

## Background on blockchain-based online social networks: The case of Steemit

Blockchain-based online social networks (BOSNs) are an emerging application of blockchain-supported technologies and present some novel and interesting characteristics which link economical aspects to online social behaviors. In this section, we introduce the architecture and the fundamental elements of BOSNs, using Steemit as a case study. We focus on Steemit as it has been one of the first and most successful platforms in the blockchain-based online social network ecosystem; and it has introduced most of the fundamental mechanisms which characterize modern BOSNs. In particular, we focus on two specific aspects, common to most of the current BOSNs: *a*) a token system based on proprietary cryptocurrency used both for fostering high-quality content and users, and supporting the validation of all social and economic actions; and *b*) a rewarding system for distributing the wealth of the platform.

Launched in 2016, the platform supports the creation and sharing of content, as well as a social network based on “follow” relationships. In Steemit, users create original blog posts, that can be shared or upvoted/downvoted by other users. Users can be *creators*—content producers—or *curator*s—content promoters. The promotion and evaluation of content are made through social actions, such as upvoting (e.g. Facebook’s like, Twitter’s heart button), downvoting (dislike), and sharing. The role of a user towards content determines how rewards are distributed. In fact, all these actions not only increase the visibility of posts but also have an economic impact. But, unlike other popular online social networks, the economic impact of these actions is explicit and measurable through the amount of gained tokens. In fact, at the end of a 7-day period, the most popular posts are awarded through cryptocurrency tokens, and both creators and curators of the most liked posts get a share of this reward. These mechanisms are inspired by the attention economy and token economy principles [2]. Indeed, active users have a financial incentive for their participation, as they are rewarded for their contributions to the platform. Rewards are distributed in the form of cryptocurrency, which can be traded among users and can be exchanged for traditional currencies like the US Dollars—USD. This way the economic value of posts and users is easily quantifiable and publicly available. This last point constitutes the pivotal link between the socio-economic dynamics internal to the platform and the external financial ones, first of all, the trend of the cryptocurrency market.

### The token system

The rewarding system, the importance—influence—of the users, and the inter/intra financial relations are mainly based on the cryptocurrency system of Steemit, which includes three different tokens, each with a specific purpose [3]: *a*) STEEM (Capitalized to avoid confusion with the Steem blockchain); *b*) Steem Dollar—(SBD); and *c*) Steem Power—SP.

**Fig 1:**
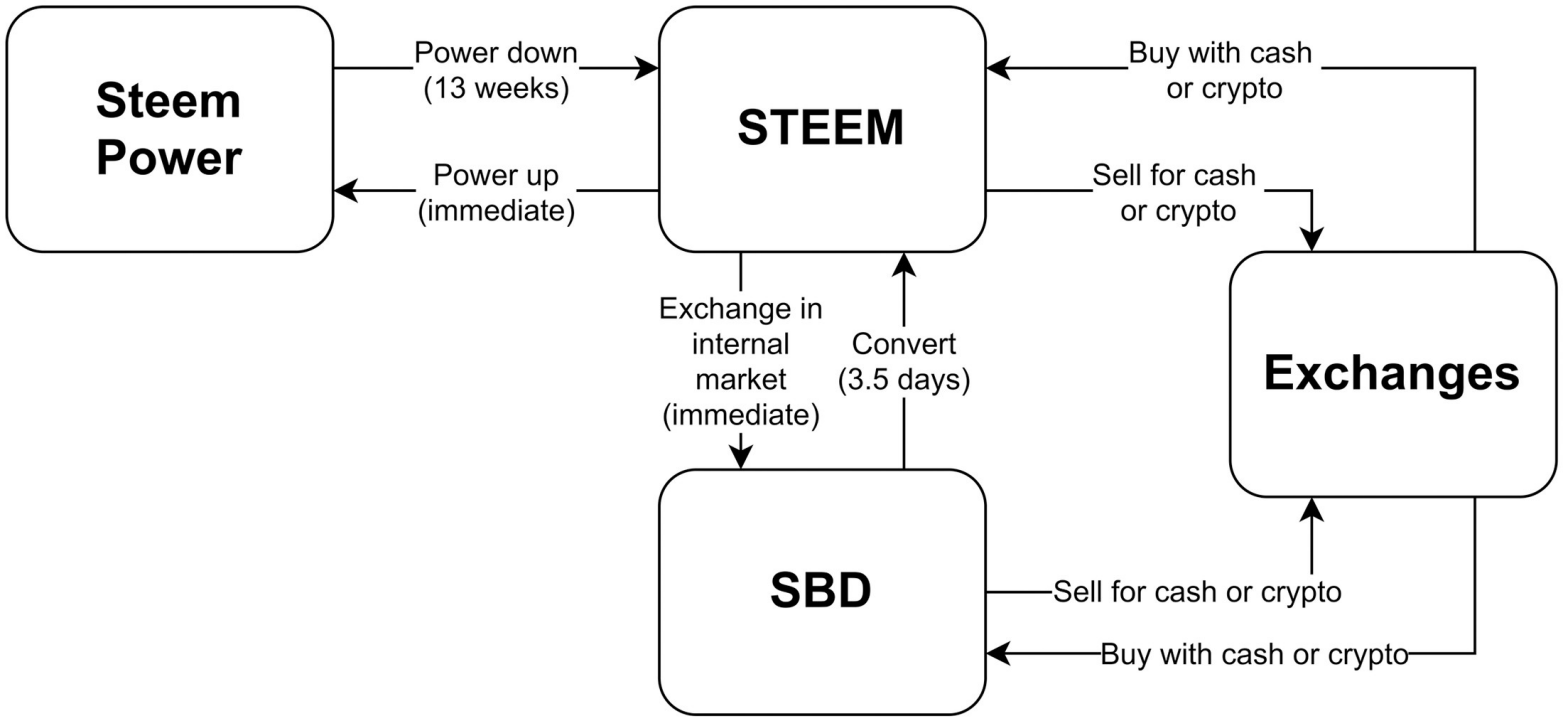
Tokens and conversion operations. Main currencies (rounded rectangles) in Steemit and possible conversion operations, are depicted as arrows. For each conversion or exchange operation, we report the type of the operation and temporal constraints, when available. In fact, many operations are instantaneous, while some others require more days.

A summary representation of the token system is shown in Fig 1, reporting the possible conversion methods as well.

The first token, STEEM, is the liquid cryptocurrency at the base of the token system. This token can be exchanged by users as a form of payment and it is tradable on different exchanges with other cryptocurrencies or more traditional currencies like US dollars. These characteristics cause the STEEM value to fluctuate. This is a key point of this study which has precisely the purpose of investigating how such fluctuations affect the usage of social actions, and, consequently, the structure of the social network. Moreover, all other tokens derive their value from the STEEM price.

As described in the Steem Blue Paper [4], there is often confusion behind the relations between tokens and their real-world values. Steem Blockchain Dollar (SBD), a coin that represents the value of STEEM as US Dollars, has been introduced to mitigate this issue and to bring a stability tool in the system. In fact, SBD, by being pegged to $1 USD, makes the economic system more accessible to newcomers: for example, rewards are usually shown as SBD. As STEEM, SBD can be bought and traded outside the Steemit platform through exchanges. It is to note that even if it was intended to be priced as $1 USD, in some periods it has reached higher values, i.e. around $13 USD.

The third currency is Steem Power (SP). This currency quantifies the amount of investment in the platform, i.e. the amount of wealth staked in the platform. Steem Power is the equivalent of market shares of Steem. As in common shares, if the value of the company increases, so does the value of users’ shares. Steem Power cannot be acquired or traded, a substantial difference from the other currencies. In short, the only way to get Steem Power is either as a reward or by converting other tokens in Steem Power.

While users are not required to spend the STEEM or SBD for participation in daily activities (e.g. posting content), they need to hold some Steem Power because the amount of Steem Power determines the number of social actions (posts, comments and votes) a user can perform. Moreover, Steem Power influences activity in the network in different ways. For example, Steem Power plays a key role in the rewarding process, as shown later: posts voted by users owning a large volume of Steem Power gain more visibility and the top posts also get a larger share of the reward pool. If a user is not interested in social activities, STEEM and SBD tokens can be exchanged for services and/or goods with other users or on trading platforms.

### The rewarding mechanism

A further central element in Steemit, and in other BOSNs, is the rewarding system, i.e. the set of rules and mechanisms regulating how Steem Power and other tokens are distributed among the users who actively participate in the platform through the production/interaction with content. The wealth distribution is based on the roles introduced so far: *creators* and *curators*. Creators publish content, either as posts or comments on posts, while content promotion—*curation*—is made through different social actions, such as upvoting, downvoting or sharing. Upvotes are key in promoting high-quality content since more upvotes give more visibility on the main pages of the platforms. Also, if the post enters into the most popular chart, the curator will gain a reward, as “users get paid for figuring out who should get paid” [3].

**Fig 2:**
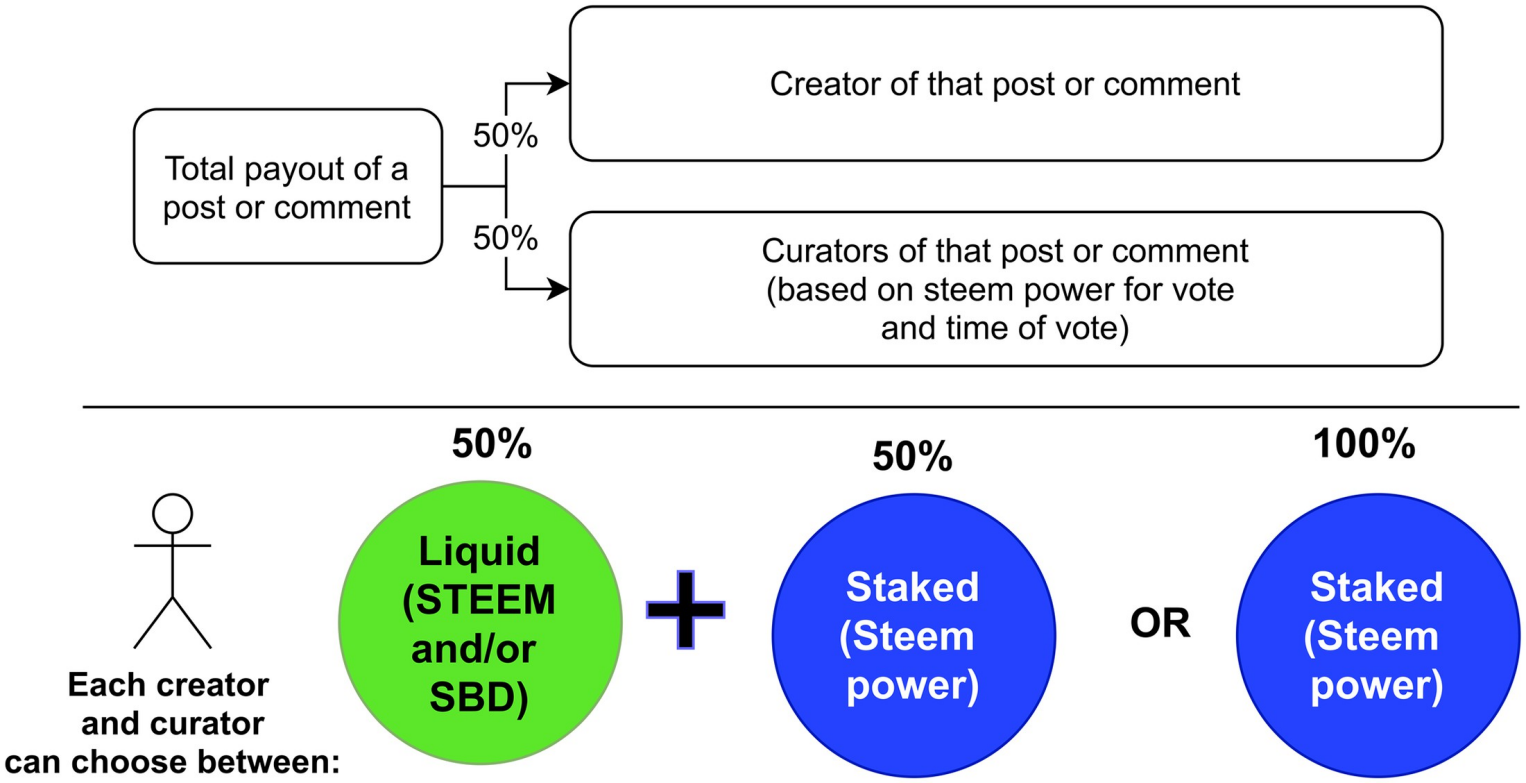
Allocation of the content payout pool. Summary of the allocation of the content payout pool between creator and curators. On top, the distribution of the rewards between the creator and curators. On the bottom, rewards can be received in two ways: full amount as Steem Power or as 50/50 split in STEEM/SBD and Steem Power.

Reward assignment is not an instant operation, in fact, rewards for content are computed after 7 days. The basic rationale behind the reward assignment procedure is that “most popular posts get more from the reward pool”. The total payout pool for a single post—content payout pool—depends on the Steem Power of the curators and how much Steem Power was used for the vote. The content payout is taken from the overall reward pool—reward to be assigned to Steemit users—which is derived from the collection of tokens produced by the Steem blockchain. Then, as summarized in Fig 2, each content payout pool is split into two parts: 50% goes to the creator and 50% to curators. Each user can decide to cash out the prize in two ways: turn the full amount in Steem Power or as 50/50 split in STEEM and Steem Power.

Note that Steem Power has a key role in the reward assignment process since it is a stake-based voting system, where vote operations are backed by the user’s Steem Power. In fact, when a curator casts a vote, s/he also has to decide how much weight to put behind a vote. Finally, as votes are not all equal the curators are not rewarded evenly: the more weight behind the vote, the bigger the reward to the voter is.

The mechanism designed for wealth distribution may have an important impact on how creators “strategically” decide which accounts to follow. In fact, different strategies based on the creation of new “follow” relationships may be adopted to get the attention of the wealthy users or to collect votes from a large volume of not very powerful users. All these network-based strategies have the general goal to gain more STEEM, that can be exchanged for traditional currencies. From this perspective, the exchange value of the STEEM cryptocurrency w.r.t. USD is equally important, since a higher price favor behaviors which collect more Steem Power and exchange it into traditional currencies.

This strict interplay among the cryptocurrency market, the network-based strategies to gain more STEEM, and the rewarding mechanism have led to the hypothesis that economical/financial factors, such as the price of the STEEM cryptocurrency, may influence the social network supported by Steemit. In this paper, we mainly focus on the validation of this hypothesis and we show some pieces of evidence which are in line with it. Second, we also deal with the strategies to gain rewards. Specifically, we focus on users who have obtained the higher amount of rewards, i.e. the most successful one: do wealthy users mostly prefer financial-oriented actions or do they produce or promote content through social actions?

## Related work

Although the research field about blockchain-based solutions and networks resulting from cryptocurrency transactions is very active in the last few years ([5–8] to cite a few studies), blockchain-based social networks (BOSNs) and their specific characteristics are not fully understood, yet. Only recently the availability of tools for querying the underlying blockchain and the increasing interest in the Web3 and its related technologies have triggered studies focused on different aspects of these large-scale intertwined complex networks. For example, Li *et al*. [9] released a dataset paper, stressing the potentiality of this network, meanwhile highlighting difficulties in extracting and processing the high volume of data produced by the platform. Other works focus on the characteristics of this innovative type of social network ([1, 10–12]). User-generated content is useful for text mining tasks [13] and bot detection ([14, 15]). There is also a growing interest in social network structure. Chonan [16] and Kim *et al*. [17] focus on the structure of Steemit social network and its characteristics. Furthermore, Guidi *et al*. [18] delve into a study of the follower–following graph, and analizye other operations in Steemit [19]. Aside from the relationships among users, [20] studies block producers (witnesses) and highlights their social impact on the platform. Other works are more focused on the economic aspects: Ciriello *et al*. [21] and Thelwall *et al*. [22] analyze the relationship between rewards and content, while Li *et al*. [23] describes and analyzes the networked structures behind the Steemit rewarding system.

Even though BOSNs may provide high and detailed volumes of temporal data, there is still limited work focused on network dynamics and temporal aspects of BOSNs. For instance, Jia *et al*. [24] focus on the diffusion of contents at a mesoscopic scale, while Ba *et al*. [25] has been the first work that has started to tackle the interplay between cryptocurrency and graph evolution. This latter study is extended by this work by taking into account all the social and financial actions and inspecting the allocation strategies of the most rewarded users. Finally, further characterization of the processes and dynamical aspects of Steemit growth has been addressed in [26].

## Materials

### Dataset

In order to carry on our investigation, we collected two types of data, internal and external to the Steemit platform: *i)* data on social and financial activities performed inside the platform, and *ii)* longitudinal data of STEEM value in the cryptocurrency market. The latter information can be retrieved from [27], a website that reports the daily value of the STEEM and SBD currencies in US Dollars and other cryptocurrencies. The prices are updated daily, allowing us to collect data for the price of the exchangeable tokens in USD for the entire observation period. In the following sections, we focus on the STEEM price, reporting in S2 Text the outcomes of our analysis using SBD trend. In fact, Steem Power has value only within the platform and is not exchangeable in external markets, and SBD, given its stablecoin nature, is less prone to fluctuations w.r.t. STEEM.

The construction of the evolution of the social interactions in Steemit is completely based on the transaction recorded in the supporting blockchain Steem. Specifically, we recovered data describing the actions made by users. Users on Steemit can perform many different actions, called *operations*. According to the official documentation [28], there are more than 50 different operations on the blockchain: an overview of these operations and a taxonomy can be consulted in [19]. The collection of these operations composes a detailed temporal dataset, that describes user activity with a time granularity of 3 seconds (the timestamp of an action is derived from its block, and a new block is produced every 3 seconds). In Steemit, every operation can be retrieved from the Steem blockchain: researchers and application developers have access to the public blockchain data through a set of public APIs, so being disintermediate from the front-end platform Steemit. We collected operations from the very first block, produced on 24th March 2016, however in the following analysis we focus on the period from December 6, 2016, up to March 18, 2020. In fact, on December 6, 2016, the regular production of STEEM started, with the release of hard fork 16, as reported in the Steemit white paper [3]. The end date—March 20, 2020—corresponds to the Hive hard fork [29], the community-driven fork that led to a split of the user base. This event was disruptive as it was originated by an internal dramatic dispute. The user base completely changed: the community was split, with some users remaining on the old blockchain, and some others moving to the new one—Hive—or acting on both, others leaving the platform. The fork had a high impact on users’ activities, too. Since the effects of the hard fork are not still clear and represent an important confounding factor, we have limited our analysis to the date of the hard fork.

In this work, we study social and financial aspects: hence, we focus on two subset of user operations: *i)* social and *ii)* financial operations. Social operations include actions that users usually do on traditional social media platforms, such as posting content or votes; while we denote as financial operations those operations designated for rewards and token management. Overall, we extract nine operations. Social actions are stored in three social operations: comment, vote and custom_json; while rewards and token related operations are stored in six operations: claim_reward_balance, transfer, transfer_to_vesting, withdraw_from_vesting, delegate_vesting_shares and convert. A full description of the aforementioned operations is presented in Table 1.

**Table 1:** List of social and financial operations. Each operation is characterized by its name, its type and a full description.

Operation	Group	Description
comment	social	A user publishes content or comment on a post
vote	social	User upvotes or downwotes. Users can vote on posts and comments
custom_json	social	A general-purpose operation designed to add new functionalities without the need for new operations. Social functionalities include: i) **“follow”** to receive updates on what other users are posting, ii) **“unfollow”** to stop following other users, iii) **“mute”** to block users from the feed in case of harassing or unwanted content, and iv) **“resteem/reblog”** to share content of another user to all the followers
claim_reward_balance	financial	User claims reward for creation or curation (amounts in STEEM and Steem Power)
transfer	financial	Transfer of the main token STEEM from an account to a “target” account
transfer_to_vesting	financial	“Power up”: convert STEEM to Steem Power at the current exchange rate
withdraw_from_vesting	financial	“Power down”. the conversion from Steem Power back to STEEM
delegate_vesting_shares	financial	Borrowing Steem Power. The Steem Power is still owned by the original account
convert	financial	Conversion from STEEM to SBD

### Methods

Our first objective is to study whether users’ behavior is influenced by the cryptocurrency system, or vice versa, if the financial system is influenced by social activities. So, we first describe the methods to highlight the possible interplay between users’ behavior, expressed by the trend of social and financial operations, and the value of the cryptocurrency supporting the BOSN. Then, we focus on the rewarding system. Here, we are interested in the preferred strategies put in place by the users to gain rewards. Specifically, we focus on users who are gaining the most from the platform, the so-called *whales*.

#### Analyzing user behavior and cryptocurrency

We deal with the influence of the cryptocurrency market on users’ behavior by investigating the interplay between the trend of the crypto-token value in the market and the social/financial activities carried out by users in the platform. To this aim, we construct the time series of the token daily price and, for each of the social and financial operations, we also build the operation time series, i.e. the number of daily activities carried out by users. A side-by-side comparison of the obtained time series enables us to highlight evidence of whether and how the cryptocurrency price impacts social and financial activities.

First, we search for potential seasonal patterns by computing the *Autocorrelation Function* (ACF). The ACF measures the linear relationship between lagged values of a time series; the resulting plot—also known as *correlogram*—shows the presence of patterns or long-term trends, and seasonal patterns [30]. Specifically, the ACF is the function of autocorrelation values *ρ*_*k*_ for every lag *k*, where *ρ*_*y*_(*k*) is defined as

$$\begin{array}{c} \rho_{y}\left(k\right)=\frac{\sum_{t=k+1}^{T}\left(y_{t}-\bar{y}\right)\left(y_{t-k}-\bar{y}\right)}{\sum_{t=1}^{T}{\left(y_{t}-\bar{y}\right)}^{2}} \end{array}$$ (1)

where *y* is a time series of average $\bar{y}$ and length *T*, and *y*_*t*−*k*_ is the lagged version of *y*. If data are trended, values of *ρ*_*y*_(*k*) will be large and positive for small lags, as closeness in time will lead to closeness in lag size [30]. So, the trended time series will have ACF with positive values that slowly decrease as the lags increase. If the times series has a seasonal trend, the values of *ρ*_*y*_(*k*) will be larger for seasonal lags (at multiples of the seasonal frequency) than for other lags. Both these phenomena can be observed when data have both trends and seasonal patterns.

After focusing on the singular time series, we will shift our attention to the link between users’ actions and the cryptocurrency price. To this aim, we measure potential correlations between each operation and token prices. We evaluate the correlation by the *Pearson Coefficient* [31]. Given two time series *x* and *y*, with average $\bar{x}$ and $\bar{y}$ respectively, we compute the *Pearson Coefficient* *ρ*(*x*, *y*) as:

$$\begin{array}{c} \rho \left(x,y\right)=\frac{\sum \left(x_{t}-\bar{x}\right)\left(y_{t}-\bar{y}\right)}{\sqrt{\sum {\left(x_{t}-\bar{x}\right)}^{2}}\sqrt{\sum {\left(y_{t}-\bar{y}\right)}^{2}}} \end{array}$$ (2)

Here, values near 1 indicate perfect correlation, values near 0 indicate the absence of cross-correlation and values towards −1 indicate perfect anti-correlation.

Finally, we measure potential lead-follow relationships between time series using the *normalized cross-correlation* measure. Given two time series *x* and *y*, the normalized cross-correlation measure [32] is similar to the correlation measure: instead of correlating *x* with *y* once, we do it multiple times, considering the time series *y*, but shifted by a series of time lags *k*. We obtain a series of different correlation values *ρ*, one for each chosen time lag *k*. In our work, we consider lags in days. This measure can be expressed as:

$$\begin{array}{c} \rho_{xy}\left(k\right)=\frac{\sum_{t=k+1}^{T}\left(x_{t}-\bar{x}\right)\left(y_{t-k}-\bar{y}\right)}{\sqrt{\sum_{t=1}^{T}{\left(x_{t}-\bar{x}\right)}^{2}}\sqrt{\sum_{t=1}^{T}{\left(y_{t}-\bar{y}\right)}^{2}}} \end{array}$$ (3)

where *x* and *y* are time of length *T*, and average $\bar{x}$ and $\bar{y}$, respectively. This calculation produces a set of pairs (lag, correlation value). We can better explore them by analyzing their shape and focusing on the time lags *k* that show the highest correlation values. If we find high correlation values for a positive time lag, then *x* leads *y*; vice versa, if the highest values are for a negative time lag, then we have that time series *y* is leading *x*.

#### Users’ behavior and rewards

In order to study the relations between users’ behavior and the gained reward, we characterize users in two dimensions. We construct for each user, a profile that summarizes two key aspects: *i*) gained rewards, and *ii*) user activity, i.e. number of actions performed.

As for the first aspect, we look at the total amount of rewards received, and select the users who have gained the most from the network. This way, we focus on users who have adopted the most effective behaviors. Operationally, in our analysis, we identify the *hubs*, i.e. the users in the top 10% of the reward distribution for each currency. The choice of considering all the currencies is platform-dependent. In the case of Steemit users can decide how to receive their rewards, so we may find different behaviors based on currency.

As for user’s activity, we look at the different types of actions provided by the platform (see Table 1) and for each user we measure *i*) whether s/he relies more on curation or creation, based on currency, and *ii*) whether s/he relies more on financial or social actions, based on currency. To this aim, each user *u* is characterized by a triple (*s*_*cr*_, *s*_*cu*_, *f*), where *s*_*cr*_ denotes the overall volume of comments and posts published by *u*, *s*_*cu*_ indicates the number of voting operations made by *u* and *f* corresponds to the total volume of financial operations. From this triple, we can measure whether an individual relies more creation or curation actions through the

$$\begin{array}{c} creation\_index=\frac{s_{cr}}{s_{cr}+s_{cu}} \end{array}$$

and the

$$\begin{array}{c} curation\_index=\frac{s_{cu}}{s_{cr}+s_{cu}}=1-creation\_index. \end{array}$$

Similarly, we can measure whether s/he relies more on social or financial actions by computing the

$$\begin{array}{c} social\_index=\frac{s_{cr}+s_{cu}}{s_{cr}+s_{cu}+f} \end{array}$$

and the

$$\begin{array}{c} financial\_index=\frac{f}{s_{cr}+s_{cu}+f}. \end{array}$$

For both measurements, higher values mean more reliance on social or financial actions, respectively. We compute these indexes for each user, then through an analysis of their distribution, we can inspect the overall behavior and potential differences between currencies. Finally, by correlation analysis, we analyze the relationships among the dimensions *s*_*cr*_, *s*_*cu*_, *f* and the rewards obtained in the three token systems.

## Results

### Interplay between users’s social/financial actions and STEEM price

In our study, we applied the above framework to study the relationship between the value of BOSN token in the market and social/financial activities of users to the case of Steemit. The methodology allows us to find evidence of a possible influence of the price of STEEM cryptocurrency on social actions provided by Steemit. As previously stated we focus on STEEM since it is the principal token in Steemit and its price heavily fluctuates due to the high volatility of the token (for the sake of completeness in S2 Text, we also reported the outcomes got by applying the above methodology on the SBD price). We analyze the time series of all the operations performed by the users of the platform, described by the number of actions per day. Alongside them, we analyze the daily price of STEEM. From the overview of all the time series, it is evident the impact the currency value has on users’ actions. The successive quantitative trend and correlation analysis reveal a significant pattern of correlations.

#### Time series: Currency and user actions

**Fig 3:**
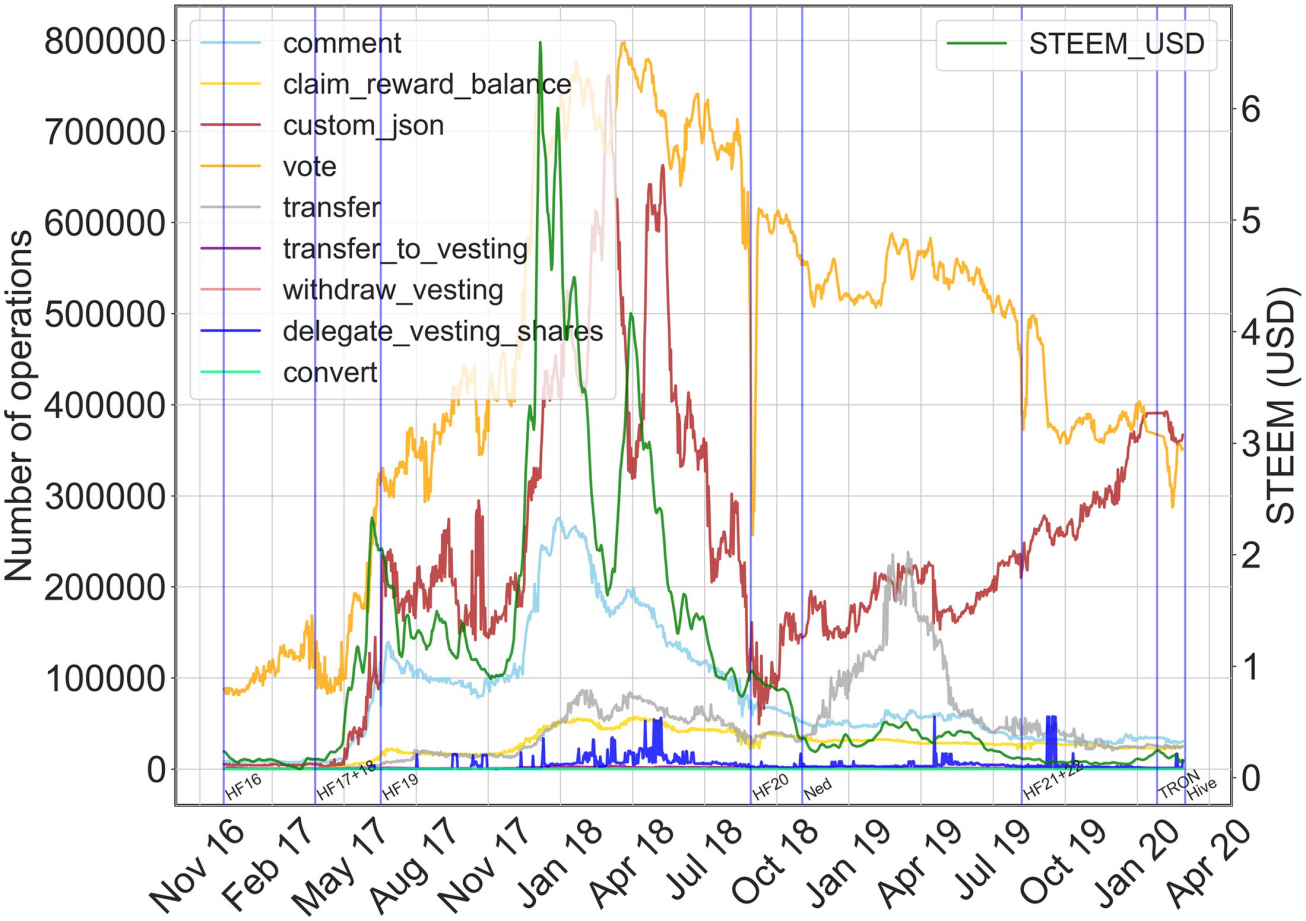
Social/Financial action time series and STEEM price. Time plots of the daily volume of social and financial operations along with the STEEM price in USD (green). On the x-axis: time in days. On the left y-axis: volume of operations per day (visualization of smoothed values, with a running average window of 7 days). On the right y-axis: STEEM price in USD. The blue vertical lines correspond to important events, like hard forks (HFXX), the crisis announcement by Scott (Ned)—Steemit founder, the selling of the company to TRON Foundation (TRON) and the Hive fork (Hive), which corresponds to the end of the observation period.

By looking at an overall picture of the time series of all the social/ financial actions and of STEEM price, we get some preliminary qualitative evidence. We displayed in Fig 3 the time series of the number of operations per day carried out by all users of the platform for the main social and financial actions, and the currency value. In the figure, we highlighted—blue vertical lines—important external or internal events that may have affected the network growth and/or the value of the STEEM currency.

As for the exchange value of the STEEM currency, we observe a few distinct phases: from November 2017 to the second half of December 2017 there is a rapid growth phase, where STEEM reached its maximum quotation; this period is followed by an equally rapid decrease until March 2018, where STEEM bounced back for a short period—April 2018. After this date, we observe a continuously decreasing trend until the end of the observation period. Per se, STEEM trend has followed the trend of other cryptocurrencies, but specific correlations may emerge if we also consider the trends of the other platform operations. For instance, if we focus on social operations only—which have reached the highest volume of actions—we can see hints of temporal correlation between social actions and cryptocurrency. In particular, the STEEM value and the volume of custom_json operation show similar traits, given a time-shift, as already detected in [23] and in [25] on “follow” relationships. For example, a first period of growth of the custom_json volume (April 2017–June 2017) corresponded to a higher STEEM price, or, more evidently, the first rapid growth of STEEM corresponded to an equally rapid increase of operations which reached the peak on March 2018; while a bounce similar to what occurred to STEEM has also happened to custom_json operations on April 2018. And again, a drop in STEEM price hampered the overall activity in the network until the hard fork 20 (HF) and the letter sent to the Steemit community by the founder Ned Scott on 28/11/18, confirming the crisis of the platform [33].

All the above considerations come from a graphical inspection of the trends; in the following, we analyze the time plots more in detail and we perform a quantitative evaluation of correlations between social/financial actions and STEEM price.

#### Trends in time series

**Fig 4:**
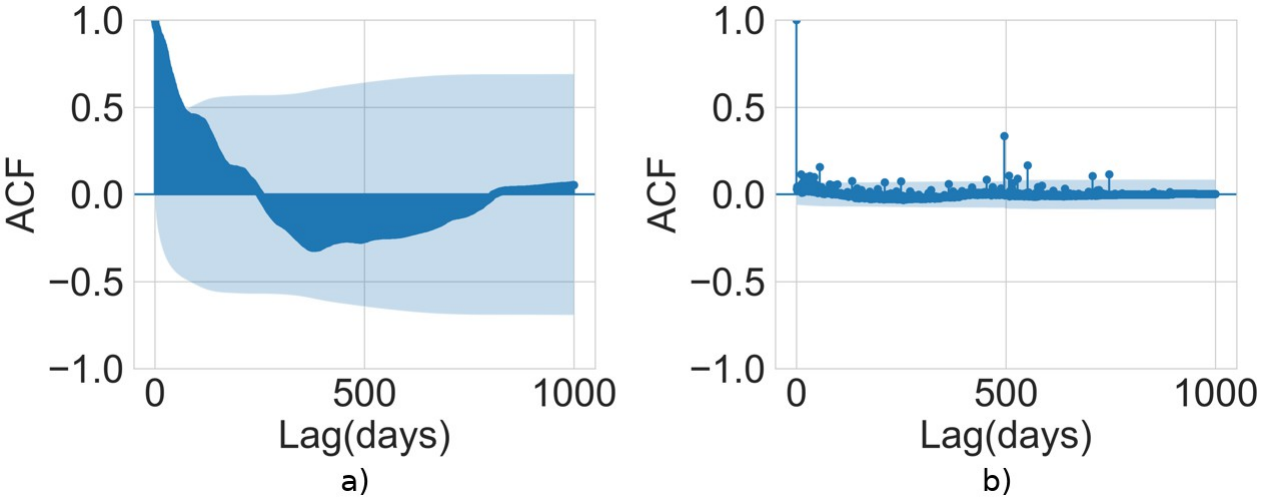
Autocorrelation Functions (ACFs). The autocorrelation function for the *a)* STEEM price and *b)* delegation of Steem Power. On the y-axis: the correlation coefficient *ρ*_*y*_(*k*). On the x-axis: the lag *k* in days. The light cyan area corresponds to the 95% confidence interval for the correlation coefficient.

A preliminary analysis has been conducted on each time series to identify if the above hints of correlation are a consequence of seasonal patterns or trends of the time series itself. In fact, a weak or missing signal of the presence of this kind of patterns would support a search for correlations among the social/financial time series and the STEEM trend. We search for seasonal patterns and trends by computing the auto-correlation function for the time series, since the resulting *correlogram* potentially shows the presence of long-term trends and seasonal patterns if exist. A subset of correlograms is reported in Fig 4, while the whole set can be found in Fig 1 in S1 Text.

The STEEM price correlogram, in Fig 4a, is characterized by the lack of repeating peaks, suggesting the absence of seasonal trends. However, the STEEM price has a short-trend since we observe positive values that slowly decrease as lags increase, but only the very first lags are characterized by a positive correlation coefficient which lies outside the confidence intervals, so statistically significant. This trait is common to other actions, showing similar characteristics, except for a few. More precisely, only two actions show different traits, namely two financial actions: lending Steem Power (delegate_vesting_shares) and powering down (withdraw_vesting). The autocorrelation plot of the former is shown in Fig 4b. Here, the main difference with the STEEM price time series is the rapid drop for small lags, a typical characteristic of time series without a trend. To sum up, all the operation time series do not show seasonal trends, and we only observe short-term correlations. This way, the hints of correlation may be searched by comparing pairs of time series.

#### Social actions and currency

**Fig 5:**
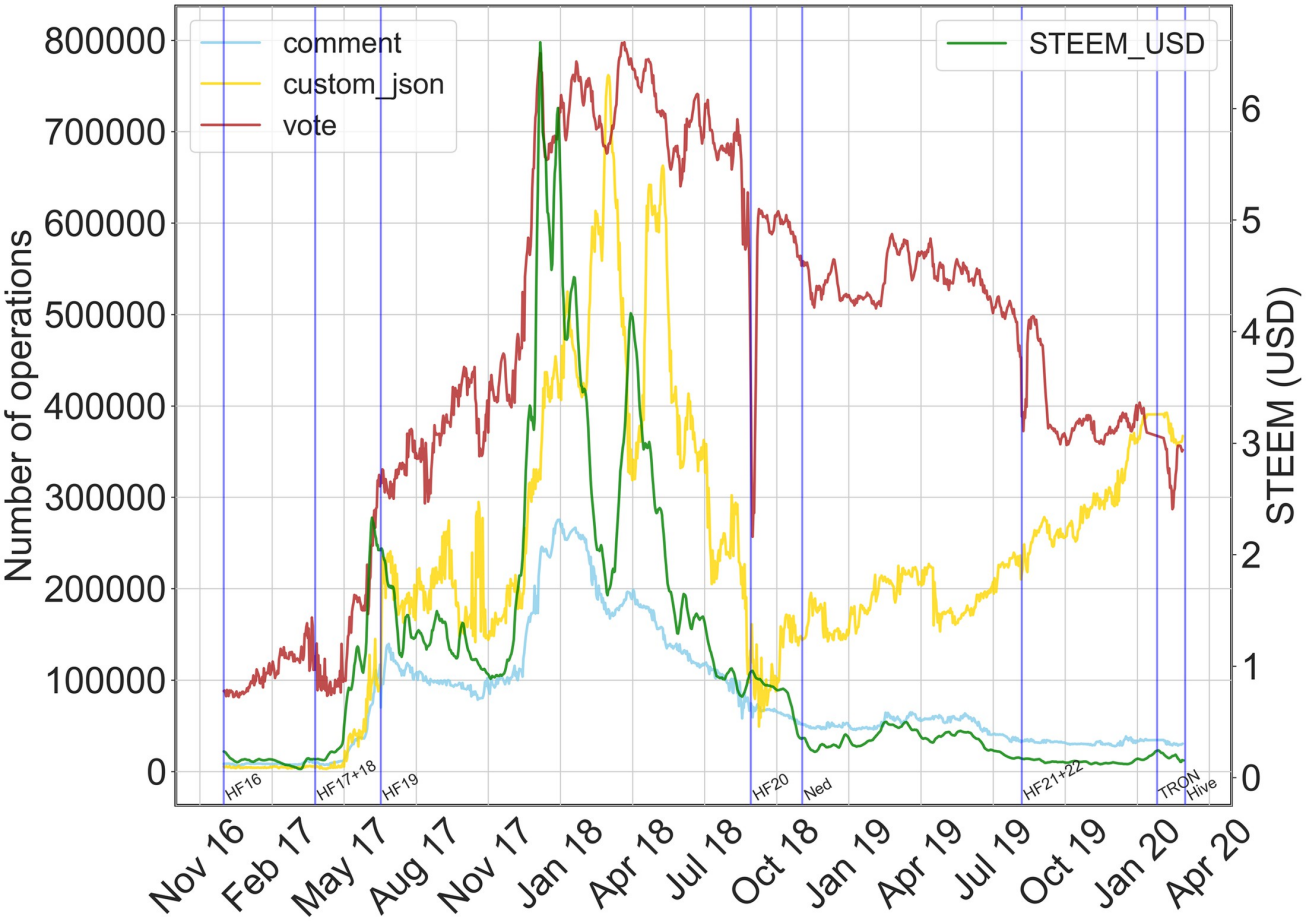
Daily volume of social actions. Time plots for social operations (vote, comment and custom_json) and STEEM price value in USD (green). On the x-axis: time in days. On the left y-axis: volume of operations per day. On the right y-axis: STEEM price in USD. The blue lines correspond to important events, like hard forks (HFXX), the crisis announcement by Scott (Ned), the selling of the company to TRON Foundation (TRON) and the Hive fork (Hive).

Since we are interested in verifying whether financial factors impact how people connect in the social platform, we first look at social actions and their relationship with the STEEM currency. In fact, social actions directly determine or are strictly related to the social graph. The time plots for the social actions—vote, comment and custom_json—and STEEM price are displayed in Fig 5, and time plots for each action can be consulted in Fig 2 in S1 Text.

First, we observe that most social actions drop in volume as time passes. The creation of posts and comments—both included in the comment operation—dropped as the currency price falls during the first quarter of 2018. As expected, the incentive to post and comment is weaker as the STEEM price is at the lowest; in fact, a user mainly interested in increasing their rewards by producing high-quality content has to spend a big effort to gain rewards with a low value. From a quantitative standpoint, we find positive correlations between STEEM price and social actions, as reported in Table 2. More precisely, STEEM value and posts/comments show a strong positive correlation (0.91). Moreover, by cross-correlation measure, we can also analyze the temporal correlation. In fact, we find an even stronger positive correlation with a maximum cross-correlation of 0.94 associated with a small lag of days, i.e. 15 days.

**Table 2:** Social actions and cross-correlations with STEEM price. The column “Total” reports the overall volume of operations during the observation period. The second column reports the average daily volume. In the last three columns we report the cross-correlation, the maximum cross-correlation and the lag with the highest cross-correlation, respectively.

Operation	Total	Average	Corr	Max XCorr	Lag (days)
comment	93832667	79654	**0.91**	**0.94**	15
vote	546677598	464073	0.53	**0.78**	97
custom_json	270860412	229932	0.52	**0.82**	40
custom_json (followed)	134608190	114268	0.74	**0.91**	36
custom_json (unfollowed)	20179192	17130	0.58	**0.80**	44
custom_json (muted)	540182	459	**0.82**	**0.92**	16
custom_json (post share)	8267940	7019	**0.87**	**0.93**	11

As for vote operations, we notice a similar drop in volume. It would be expected, as there is less content to consume and, especially after the hard fork 19—HF19 -, the voting power is limited by the amount of Steem Power owned by voters. However, the drop is not as marked as we see in comments, which is reasonable, as votes still require less effort than producing content; in fact, users only need Steem Powers and a click on the post/comment. Therefore the correlation between votes and STEEM price is much lower—0.53—than the comment correlation and a moderately positive cross-correlation can only be found with a high lag of more than 90 days (see Table 2).

custom_json operation has a different evolution: in the initial period, until the hard fork HF20, the number of operations behaves more like posts and comments, rising and dropping as STEEM price does. However, after the hard fork H20, we can observe that the number of daily operations started an increasing trend again. In this case, this is a consequence of the fact that among the operations we have not only social actions (follow, share, unfollow, ignore), but also other actions, as well. In fact, new apps and platforms can rely on custom_json operation to save their data. These operations are also used by other services outside Steemit, other decentralized apps, such as Dtube (https://d.tube) and SteemMonsters (https://splinterlands.com).

To understand whether the rise is caused by social actions or other factors, we detailed the custom json time series by separately analyzing the daily volume of the specific actions contained in custom json records (see Figs 3–5 in S1 Text). The key takeaway is that among the operations belonging to custom_json category, social actions (follow, share, unfollow, ignore) have declined as the other social actions (votes, posts, comments), so the trend after HF20 is mainly driven by new operations performed by decentralized apps operating on the Steem blockchain. Thus, we observe a clear shift in how the blockchain is being used (HF20 has introduced many changes; among them, a revamped system, currently in use, that influences the number of actions allowed for a user).

Given the above observations, we separately measure the cross-correlation between the STEEM price and the social actions in custom_json operations, and the STEEM price and the other actions in custom_json. In fact, by isolating the main social actions (follow, share, unfollow, ignore), we obtain much higher values of cross-correlation, with lower day lags, with respect to correlations and lags computed comparing the STEEM price and the overall volume of custom_json operations, as shown in Table 2.

In general, the analysis of the cross-correlations among the STEEM price and the different social actions highlights that cryptocurrency had an impact across all social activities. This represents a first evidence of a possible influence of economic and financial factors on the structure of the social graph supported by blockchain-based online social networks.

#### Financial actions and currency

**Fig 6:**
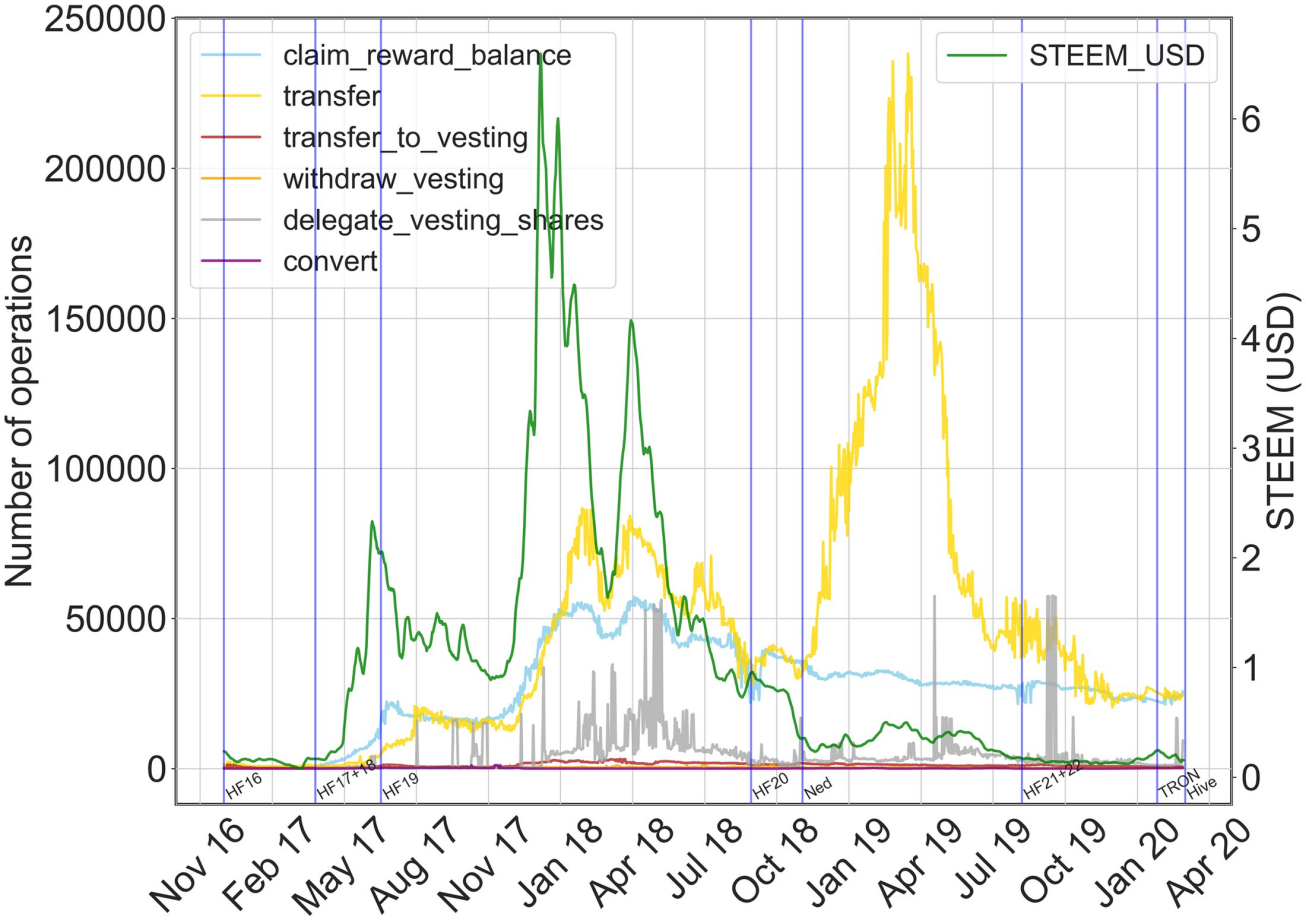
Daily volume of financial actions. Time plots for financial operations and STEEM price value in USD (green line). On the x-axis: time in days. On the left y-axis: daily volume of operations. On the right y-axis: STEEM price in USD. Blue vertical lines correspond to important events, like hard forks (HFXX), the crisis announcement by Scott (Ned), the selling of the company to TRON Foundation (TRON), and the Hive fork (Hive).

We also focused on the relation between the STEEM price and financial actions in Steemit, since most of them determine an interaction between Steemit users. These actions are less frequent in the network, in terms of daily volume, with respect to social actions but still notable since they represent an element of novelty in the online social network landscape. Following the above methodological approach, we first look at their daily volume, as reported in Fig 6, where we jointly display their time plots (plots for each action can be consulted in the Fig 2 in S1 Text). Alongside these plots, we report volumes and correlation measures in Table 3, respectively.

**Table 3:** Financial actions and cross-correlations with STEEM price. The column “Total” reports the overall volume of operations during the observation period. The second column reports the average daily volume. In the last three columns we report the cross-correlation, the maximum cross-correlation and the lag with the highest cross-correlation, respectively.

operation	Total	Average	Corr	Max XCorr	Lag(days)
claim reward balance	31609874	29709	0.53	0.80	26
transfer	55033746	46718	0.04	0.83	436
transfer to vesting	1393465	1183	0.56	0.80	39
withdraw vesting	344062	292	0.30	0.60	93
delegate vesting shares	5260366	5039	0.12	0.33	90
convert operation	101308	93	-0.02	0.46	-140

As observed in the case of social actions, we can see that most financial actions dropped in volume as the value of the currency dropped. However, time series have different traits, mainly due to the type of currency involved in the action. For example, reward claims (blue line) are not dropping as steadily, as the reduction of user activity results in less competition. While the amount of conversions to Steem Power (transfer_to_vesting—red line), which are the equivalent of an investment in the platform, drops as the currency becomes less valuable. For these two types of financial actions, we observe a medium positive correlation (0.53 and 0.56), while getting their maximum cross-correlation after about 30 days (0.8 and 0.83), respectively. Whereas other operations seem to have a weak relationship with the STEEM price. For example, we can observe that the power down operation (withdraw vesting), has spiked in the crisis periods. Similarly, the conversion to SBD (convert) reached its lowest during the period of crisis, as users were looking to move back to STEEM to trade and cut losses or to try and speculate. In these cases, we observe weak correlations and cross-correlation with lag values too high to be related to a possible influence, especially for convert operation.

We also obtain a low correlation value for transfers of STEEM, even if its trait is different from the other financial actions: while they seem to rise and fall as the other actions during the first half of the observation period, we notice a spike after the hard fork HF20. While the cross-correlation value is high, the lag is too long—436 days, indicating a not-informative correlation.

Finally, we observe some spikes in lending of Steem Power (delegate_vesting_share), that are not related to STEEM price: they may be related to other events where Steem Power is critical, such as witness election. In fact, the correlation values are low, suggesting that there could be other factors in play.

To sum up, the correlation values on the overall time period between STEEM price and financial actions are not as strong as in the case of social actions. While visual evidence suggests some effects, it looks like the relationship may be more complex, and deserves further analyses.

### Rewards and users: The behaviors of highly rewarded accounts

We are interested in users’ preferred ways to gain rewards in the platform. Specifically, we analyze the users with the highest rewards, i.e. the hubs or richest nodes, by focusing on *a*) whether they rely more on curation or creation based on the type of token used to claim their rewards; and *b*) whether they rely more on financial or social actions, based on the type of token. As detailed in Section, each user can decide to cash out a reward in two ways: turn the full amount in Steem Power or as 50/50 split in STEEM and Steem Power. Therefore, for each reward, we have a mix of tokens, and each time users can decide a different split. Therefore, a user might be a hub for one type of token, only. In fact, only 27% of the union of all hubs are hubs for the three tokens, while 18% are hubs exclusively for the Steem Power token, for example.

Therefore for the study of rewards, we describe a user by *a*) *rewards_sbd*, i.e. the total amount of rewards in SBD; *b*) *rewards_steem*, i.e. the total amount of rewards in STEEM; and *c*) *rewards_sp*, i.e. the total amount of rewards in Steem Power. While for user activity, we examine: *a*) the *creation* activity, i.e. the number of posts and comments; *b*) the *curation* activity, i.e. the number of votes; *c*) the *social* activity given by the total amount of creation and curation actions; and *d*) the *financial* activity, i.e. the total amount of financial actions.

The combination of these variables allows us to characterize the behavior of users, looking for potential differences in users’ activity according to the type of currency.

#### Creation and curation activity

**Fig 7:**
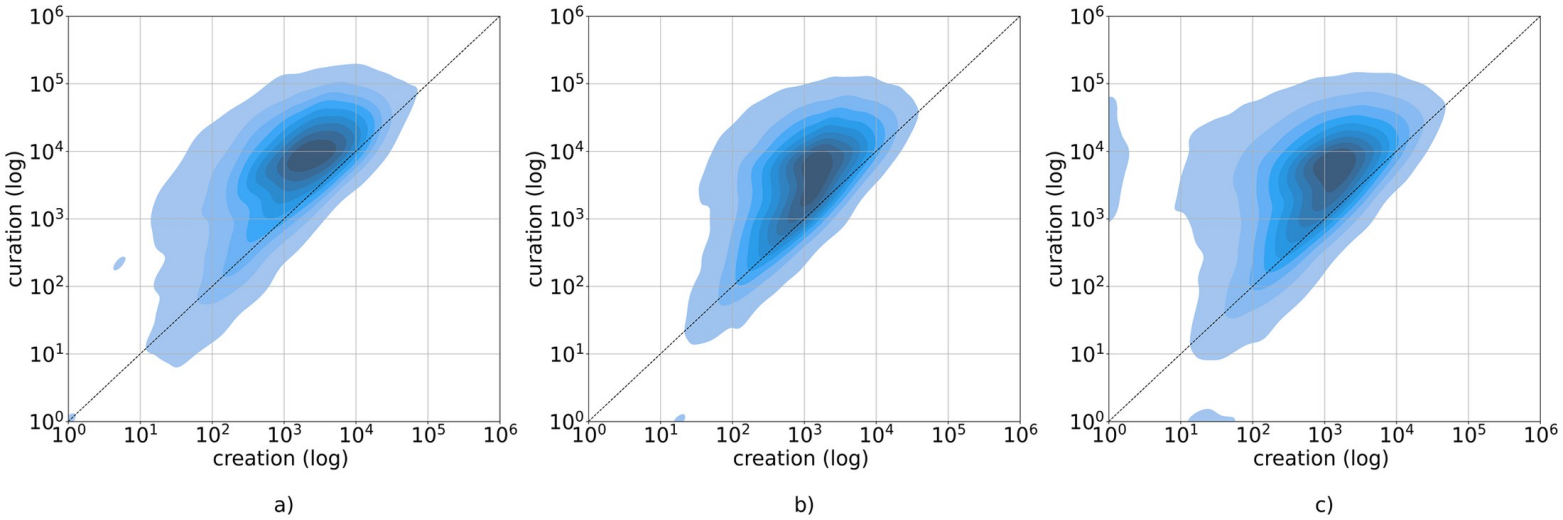
User’s creation and curation, for each currency. Scatter plots (obtained by Kernel Density Estimation—KDE) relating curation (number of votes) and creation (number of posts and comments), for each of the three currencies: a) STEEM, b) SBD and 3) Steem Power. On the x-axis: the creation activity. On the y-axis: the curation activity. Darker colored areas correspond to higher density.

We visualize the values of curation and creation for hubs in Fig 7. Through scatter plots, we can see that there is a skew toward either creation or curation for the hubs. The relation between creation and curation activities has been reported for different currencies, since users can choose how to get their rewards, either as 100% Steem Power or a 50/50 split in Steem Power and one of the liquid currencies STEEM/SBD (see Section “The rewarding mechanism”). The visual analysis shows a different distribution between Steem Power and the other currencies. In the scatter plot representing the hubs for Steem Power (rewards_vests)—Fig 7c—users are distributed in a slightly different way: we can see that there are more hubs that have high levels of curation actions, and some of them have a very low creation activity. This difference between liquid currency holders and Steem Power holders is consistent with the purpose of the tokens. Indeed, users with high Steem Power have more influence on the curation process and rewards. In fact, as the Steem Power behind the vote influences which posts become more visible and the rewards for curators are proportional to the power to their weight, it becomes more effective for Steem Power owners to curate, instead of spending time and effort creating new content.

**Fig 8:**
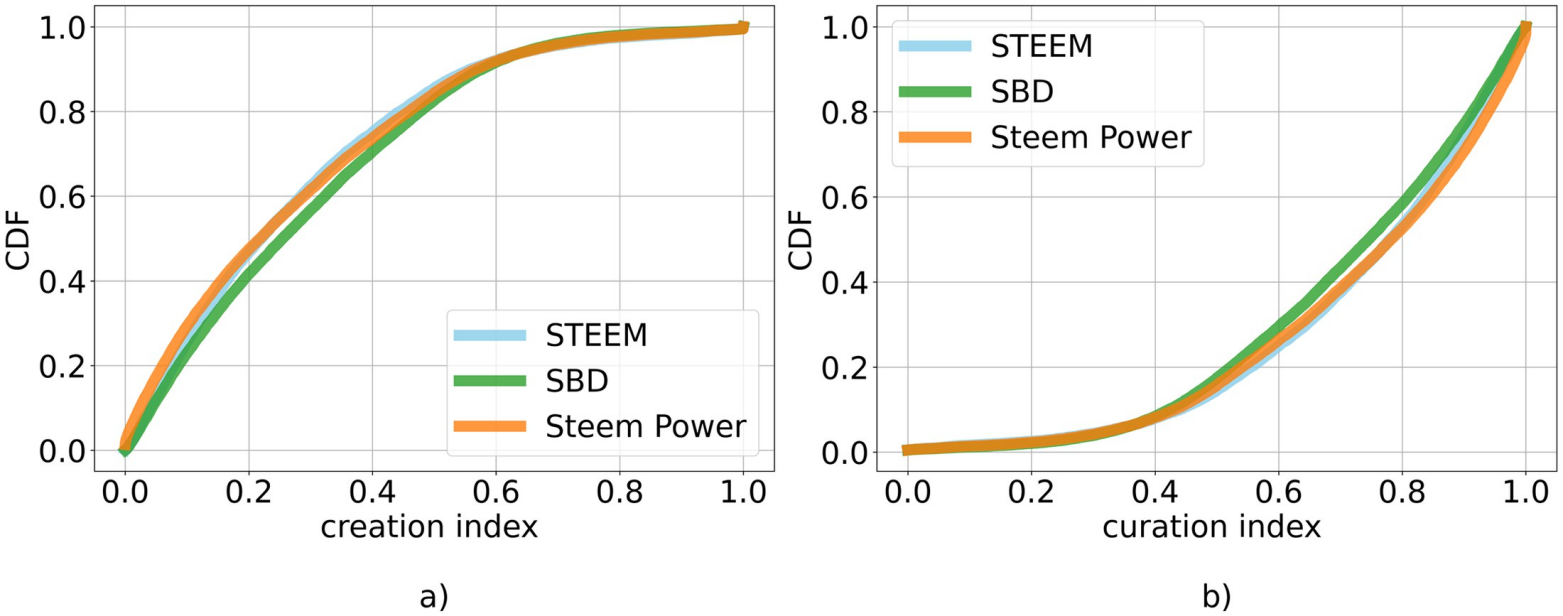
Curation index distribution. Cumulative Distribution Function of the *curation*_*index*, for each of the three currencies. On the x-axis: the creation index, which measures how much a user relies more on curation operations. On y-axis: Cumulative Distribution Function—CDF.

As for the relation between curation and creation activities, we also analyzed the *curation*_*index* and the *creation*_*index* as defined in Section Methods. In Fig 8, we visualize the distribution of the curation index for the hubs. Since the creation index is complementary to the curation index, we only report the latter. The curation index distribution shows that overall users rely more on curation, in line with the previous observation. We can also see that there are only small differences between currencies.

#### Social and financial activities

**Fig 9:**
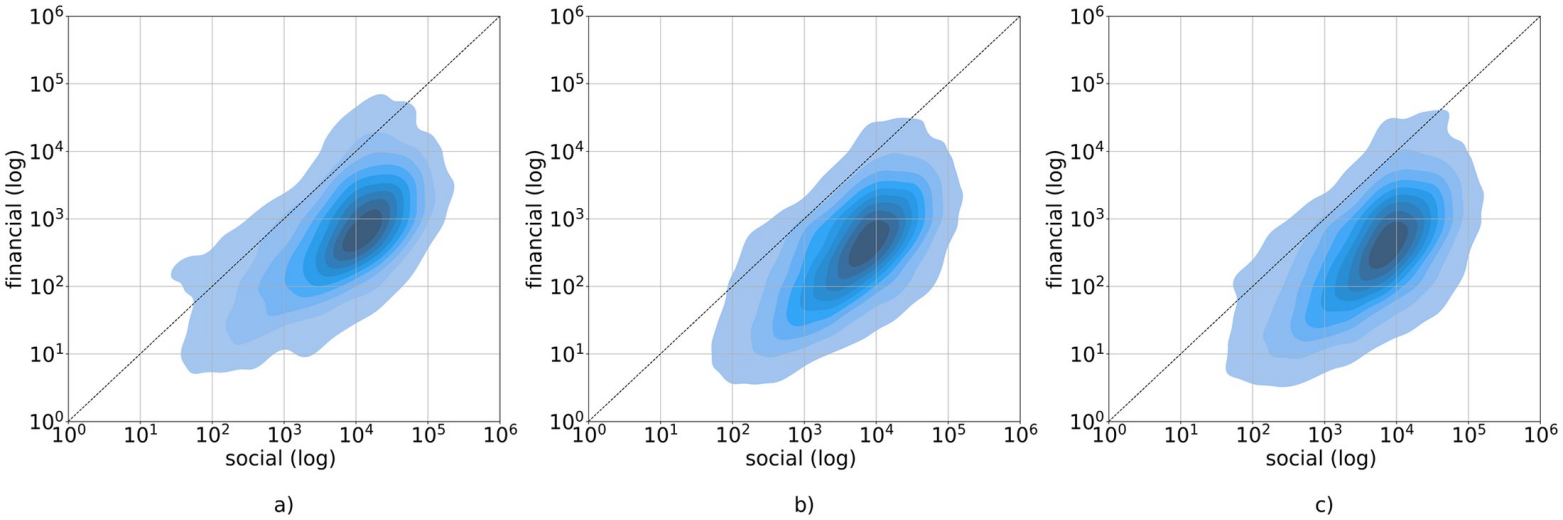
User’s social and financial activity, for each currency. KDE scatter plots relating social and financial activities (number of votes) and creation (number of posts and comments)for each of the three currencies. On the x-axis: social actions. On y-axis: financial actions. Darker colored areas correspond to higher density.

We have also applied the above approach by focusing on the relation between social and financial activities. Indeed, the allocation of social and financial actions represents a further strategy for hubs acting within the rewarding system. In Fig 9 we report the relationship between the total amount of social and financial actions for the hubs separately identified for the three types of token.

**Fig 10:**
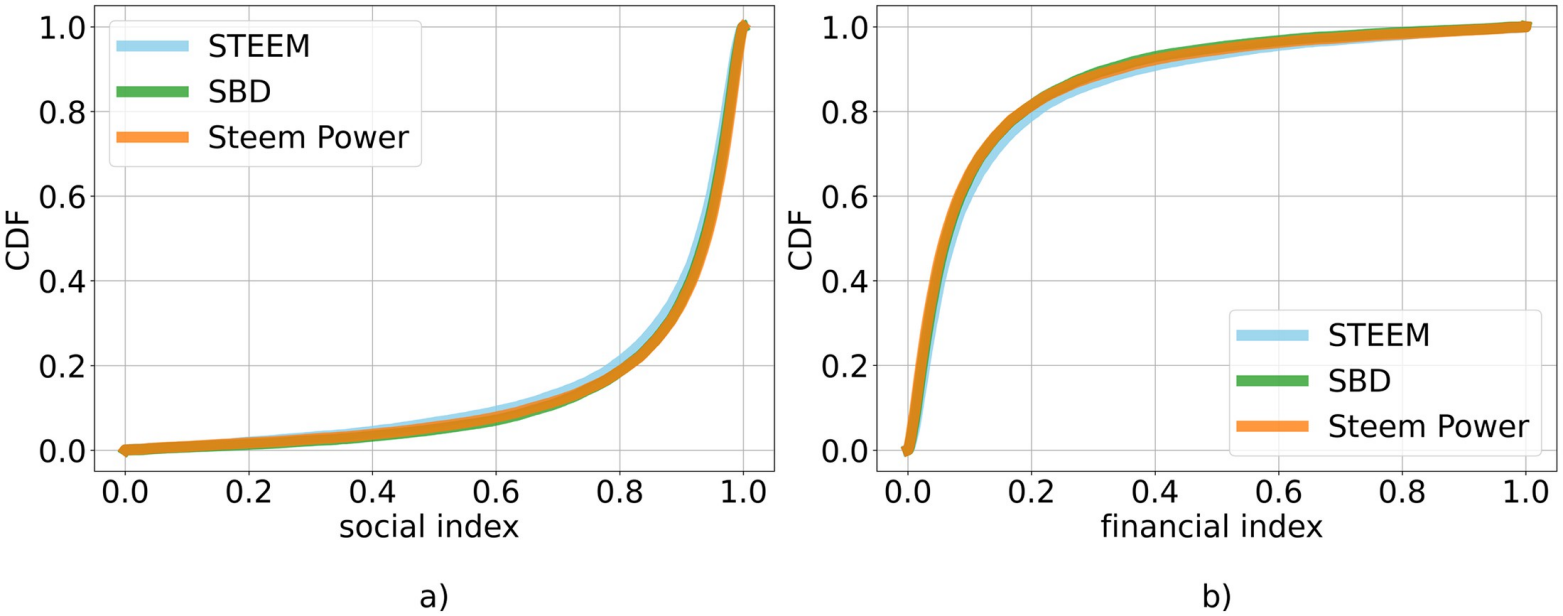
Social and financial indexes distribution. Cumulative Distribution Function—CDF—of a) *social*_*index* and b) *financial*_*index*, for each currency. On the x-axis: *social*_*index* and *financial*_*index*, that measure whether a user relies more on social or financial actions, respectively. On y-axis: CDF.

Through scatter plots we can see if there is a skew towards either one of them. As for the previous case, we look at differences between currencies. Here the difference is less marked between the currencies. Then, we visualize the distributions of social and financial indexes for the hubs in Fig 10. The distributions of the two indexes show that users mainly rely more on social actions to gain tokens. This observation is in line with the previous one, but we do not observe differences between different currencies.

#### Correlation analysis

Finally, we conducted an analysis of correlation between the rewards and the above four indexes: *creation*, *curation*, *social* and *financial*. The outcome of the analysis has been reported in Table 4, where we take into account the rewards gained through different tokens, separately. In general, we observe low correlation values across the different combinations of indexes and rewards. In fact, we find an absence of correlation with the creation factor, for all tokens. If we observe the curation operations, the correlation is higher than in the previous case, but correlation coefficients are still low. That is in line with the above observations on the scatter plots, confirming the usage of curation operations to gain rewards by some hubs. We have a similar situation when we consider the correlation values between social and financial actions: correlation values are close to zero, suggesting a lack of linear correlation.

**Table 4:** User reward hubs. Correlation of currency and indexes.

operation	STEEM	SBD	Steem Power
creation	0.07	0.08	0.08
curation	0.15	0.22	0.24
social	0.13	0.18	0.20
financial	0.02	0.03	0.11

## Discussion and conclusions

The idea of Web 3.0 revolved around decentralization solutions is becoming one of the most promising responses to the over-centralization of Web 2.0. In Web 3.0 the decentralization is mainly reached through different blockchain technologies which aim at supporting nowadays online services and platforms and promoting novel paradigms such as decentralized finance—DeFi—or self-sovereign identity. Online social networks and media, services leading the Web 2.0 landscape, are now moving towards decentralized solutions as highlighted by the rising of many different blockchain-based social networks—BOSNs. These social platforms replicate all the social functionalities which have facilitated online relationships in the past, meanwhile introducing novel mechanisms to overcome issues related to data monetization, content quality, misinformation, and censorship. As for the former two points, blockchain technologies are strongly coupled with rewarding and voting systems that, from one side, allow users to gain rewards from their content and, at the same time, promote the creation of high-quality content. In the BOSN landscape, Steemit is the seminal project and was one of the most widespread platforms. In fact, it includes the most representative features of blockchain-based social networks: *a*) contents are evaluated by users through social actions; *b*) the rewarding system incentives the production and the promotion of high quality or highly appreciated contents; and *c*) rewards are paid with an exchangeable cryptocurrency, whose value can fluctuate over time.

The above features make Steemit, and in general BOSN, a complex cyber-physical system where social, economic, and financial layers are strictly intertwined and influence each other. Indeed, users’ social actions also have an economic explicit impact measurable through the amount of gained tokens. This strict interplay among the cryptocurrency market, the network-based strategies to gain more STEEM and the rewarding mechanism has led to the hypothesis that economical/financial factors, such as the price of the STEEM cryptocurrency, may influence the social network supported by Steemit. In fact, we found evidence of the influence of the STEEM cryptocurrency over users’ actions. More precisely, we found higher values of correlation for social actions, but lower impact on financial actions. Among social actions, the cryptocurrency price strongly correlates with operations—such as “follow” or link creation—which shape the structure of the Steemit social network. So, we can reasonably state that Steemit social network has been partly shaped and driven by the trend of its cryptocurrency through the rewarding and voting mechanisms the platform has implemented. On the contrary, we do not observe a great influence of user actions on the cryptocurrency value, since correlation lags between STEEM cryptocurrency and actions are always positive. So, users seem to adapt their behavior to the cryptocurrency, whereas it seems that external events have more influence on the cryptocurrency, e.g. the price of Bitcoin.

As we have seen that the cryptocurrency value has influence over actions, we tried to detect trends or characteristic behavior of users. To this aim, we focused on hubs—the most successful users in terms of rewards—trying to understand their strategies to gain more rewards. We found some differences in users’ social behavior: hubs for Steem Power show differences in the behavior, with higher levels of curation actions w.r.t. the other currencies offered by Steemit. The difference is in line with the purpose of the tokens: it becomes more effective for those who possess more Steem Power to curate, instead of spending time and effort creating new content. However, we did not find significant differences among currencies, when we considered social and financial actions. It is interesting to notice that the purpose of the currency seems to influence user behavior, suggesting that the type of currency should be considered in the analysis.

To sum up, while this work does consider only one platform, it would definitely be interesting to extend the study to more platforms. Indeed, the analysis of rewards focuses on the hubs, but it could be interesting to explore the decisions of other categories, i.e. most influential users in the social side or people mostly involved in data validation—witnesses—and rule-making process—project developers. Despite these limitations, we were able to obtain important insights on the interplay of cryptocurrency and network activity: we showed that external events and cryptocurrency value have a strong impact on users’ activity and behavior. These insights lead to other research questions, as there is still limited understanding of the impact of external events on users’ activity and social network evolution. The study of network evolution with external events and co-evolution of networks can be improved by focusing on currency-related events, cryptocurrency growth and drop, news or announcements related to currency, in addition to disruptive events like hard forks.

## Supporting information

S1 Text Additional times series and figures. (PDF)

S2 Text Correlation analysis between SBD and social/financial actions. (PDF)
