# Supplementary material for: The role of cryptocurrency in the dynamics of blockchain-based social networks: The case of Steemit
Source: PLoS One. 2022 Jun 16;17(6):e0267612. doi: 10.1371/journal.pone.0267612 (PMC9202840; doi:10.1371/journal.pone.0267612)
Supplement: S1 Text — (PDF) [file pone.0267612.s001.pdf]

S1 - The role of cryptocurrency in the dynamics of blockchain-based social networks: the case of Steemit

Cheick Tidiane Ba [1], Matteo Zignani [1*], Sabrina Gaito [1]

1 CONNETS Lab, Computer Science Department - Università degli Studi di Milano, Via Celoria 18, Milan, Italy,

* matteo.zignani@unimi.it

1 S1 Text

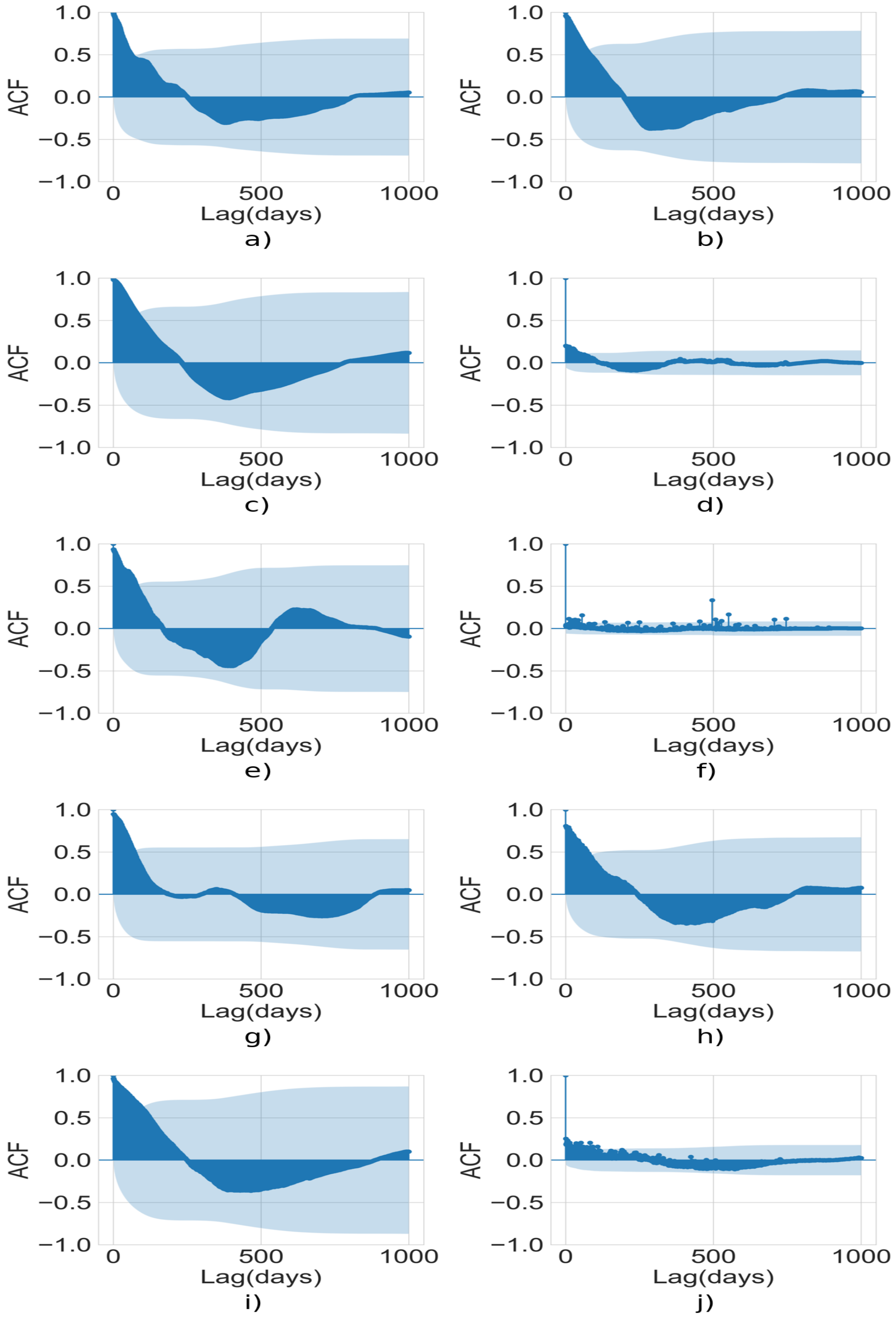

Fig 1. S1 Text. Auto-correlation functions of the operations. Auto-correlation function of the time series related to the daily volume of the following operations: a) `claim_reward_balance`, b) `comment`, c) `convert`, d) `custom_json`, e) `delegate_vesting_shares`, f) `transfer`, g) `transfer_to_vesting`, h) `vote`, and i) `withdraw_vesting`.

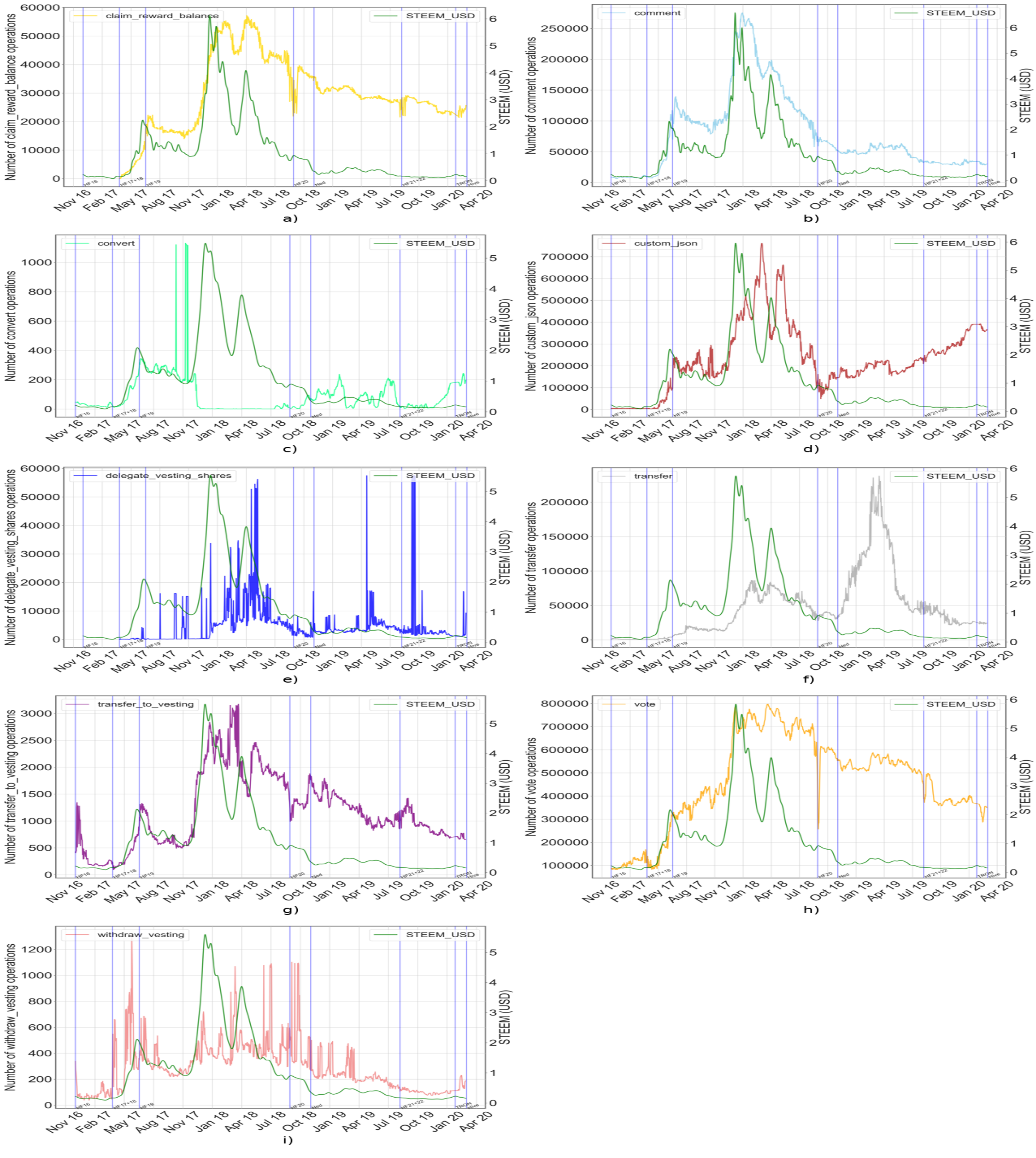

Fig 2. S1 Text. Daily volume of the operations. Time series of the daily volume of STEEM price and each of the 9 operations: a) claim_reward_balance, b) comment, c) convert, d) custom_json, e) delegate_vesting_shares, f) transfer, g) transfer_to_vesting, h) vote, i) withdraw_vesting (visualization of smoothed values, with running average window of 7 days).

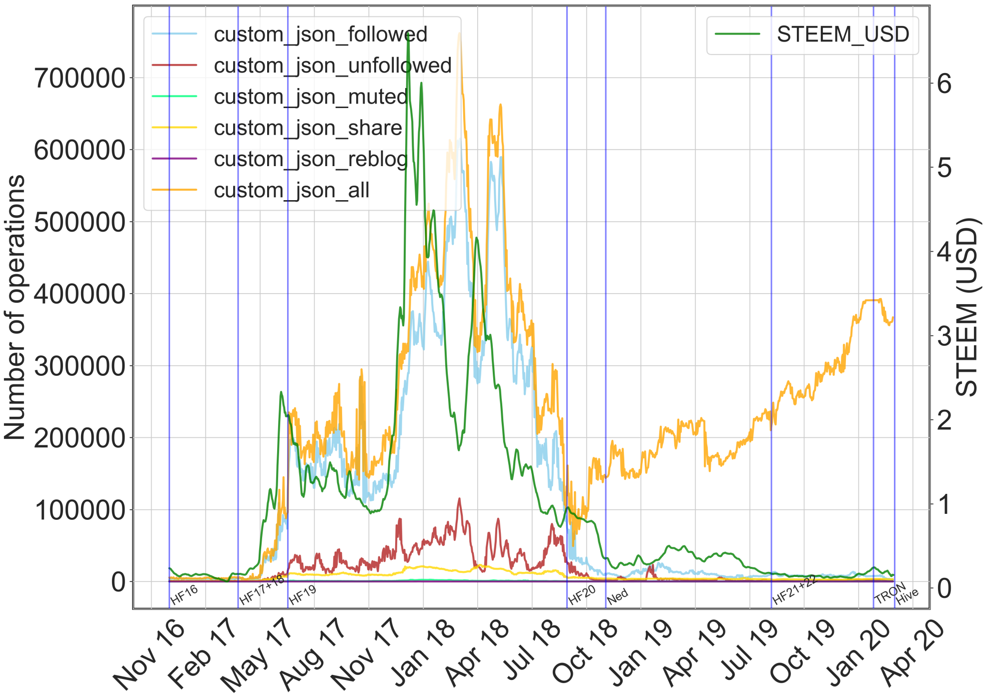

Fig 3. S1 Text. Daily volume of the social actions in `custom_json`. Time series of the main social actions included in the `custom_json` operations: follow, unfollow, mute, share and reblog (retweet): along with the STEEM price in USD (green). On the x-axis: time in days. On the left y-axis: volume of actions per day (visualization of smoothed values, with running average window of 7 days). On the right y-axis: STEEM price in USD. The blue lines correspond to important events, like hard forks (HFXX), the crisis announcement by Scott (Ned), the selling of the company to TRON Foundation (TRON), and the Hive fork (Hive).

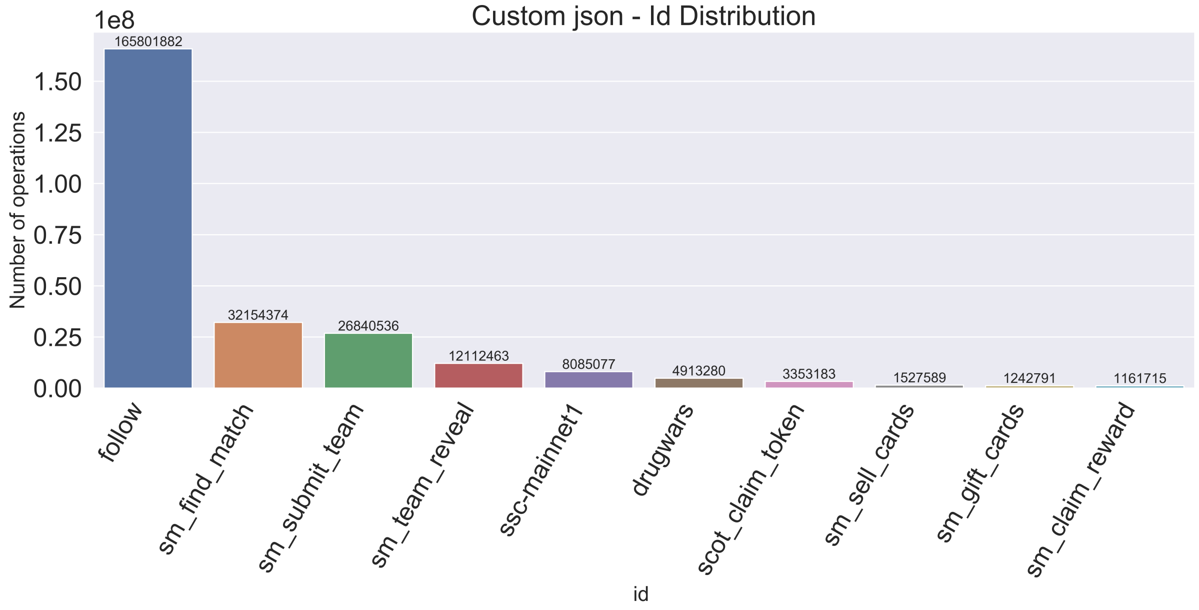

Fig 4. S1 Text. Aggregated volume of the actions included in the `custom_json` record. Number of operations for each specific action contained in `cstom_json` operations until the hard fork HF20.

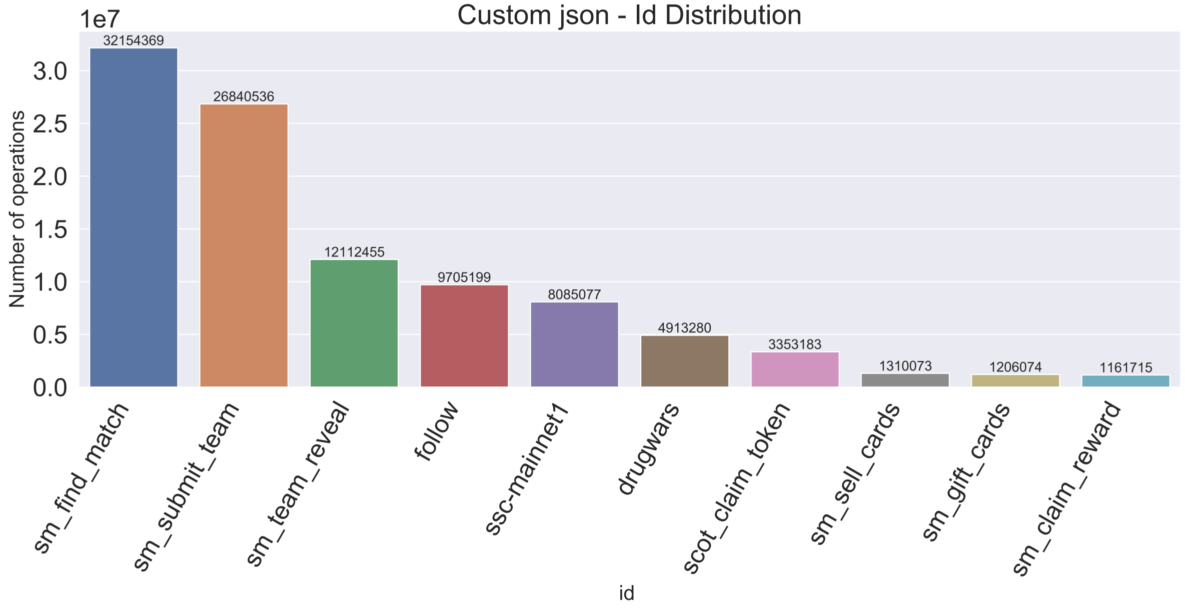

Fig 5. S1 Text. Aggregated volume of the actions included in the `custom_json` record. Number of operations for each specific action contained in `cstom_json` operations after the hard fork HF20. We observe a significant drop in the volume of "follow" actions.
