# Supplementary material for: The role of cryptocurrency in the dynamics of blockchain-based social networks: The case of Steemit
Source: PLoS One. 2022 Jun 16;17(6):e0267612. doi: 10.1371/journal.pone.0267612 (PMC9202840; doi:10.1371/journal.pone.0267612)
Supplement: S2 Text — (PDF) [file pone.0267612.s002.pdf]

S2 - The role of cryptocurrency in the dynamics of blockchain-based social networks: the case of Steemit

Cheick Tidiane Ba [1], Matteo Zignani [1*], Sabrina Gaito [1]

1 CONNETS Lab, Computer Science Department - Università degli Studi di Milano, Via Celoria 18, Milan, Italy,

* matteo.zignani@unimi.it

1 S2 Text

Since its introduction in Steemit, the Steem Blockchain Dollar - SBD - represents a stablecoin which, by leveraging the policies introduced in the original Steemit whitepaper, should have been pledged to $1 USD. However, as shown in Fig 1 its price has experienced a strong instability period from November 2017 to May 2018, mainly due to a similar instability period of STEEM, which is generally more volatile. In fact, in the case of prices greater than $1 USD, it is proven to be more difficult to bring the SBD value close to 1 because it implies forcing users to do more social actions. In this Support Information, we applied the analysis framework presented in the main paper to the SBD trend, and we report the main outcomes concerning the correlation between SBD and social actions.

Time series: currency and user actions In Fig 1 we reported the time series of the number of operations per day carried out by all users for the main social and financial actions, and the SBD value. In the figure, we highlighted - blue vertical lines - important external or internal events that may have affected the network growth and/or the SBD value. As already mentioned, we observe two different periods where the SBD price is not pledged to $1 USD: from March 2017 to June 2017, and from November 2017 to May 2018. These two periods are strongly linked to the trend of the STEEM price: during the second half of December 2017, STEEM reached its maximum quotation. In particular, SBD reached its maximum value when STEEM reached its peak, but the mitigation mechanisms for keeping the SBD stable canceled the effects of the STEEM rebound in March 2018.

Social actions and currency We compute the cross-correlation between the time series of the SBD price and each social action, separately. In Table 1, we reported, for each action, the correlation, the maximum cross-correlation and the lag corresponding to the maximum cross-correlation. As in the STEEM case, we observed a positive correlation between the token price and posts/comments; and an even stronger positive correlation with a maximum cross-correlation of 0.84 associated with a lag of 35 days. Generally speaking, we confirm a correlation trait similar to the STEEM case, with correlation values a bit less strong and larger time lags. Specifically, a strong cross-correlation between the SBD value and the “follow” operations still persists.

Financial actions and currency We also computed the correlation, the maximum cross-correlation and its time lag between the SBD price and the financial actions. The results, reported in Table 2, show a correlation and lag values comparable to the STEEM analysis in the paper.

Time series of social and financial actions For the sake of completeness we also displayed the time series of the different financial actions (see Fig 2) and social actions inside the `custom_json` operations (see Fig 3)

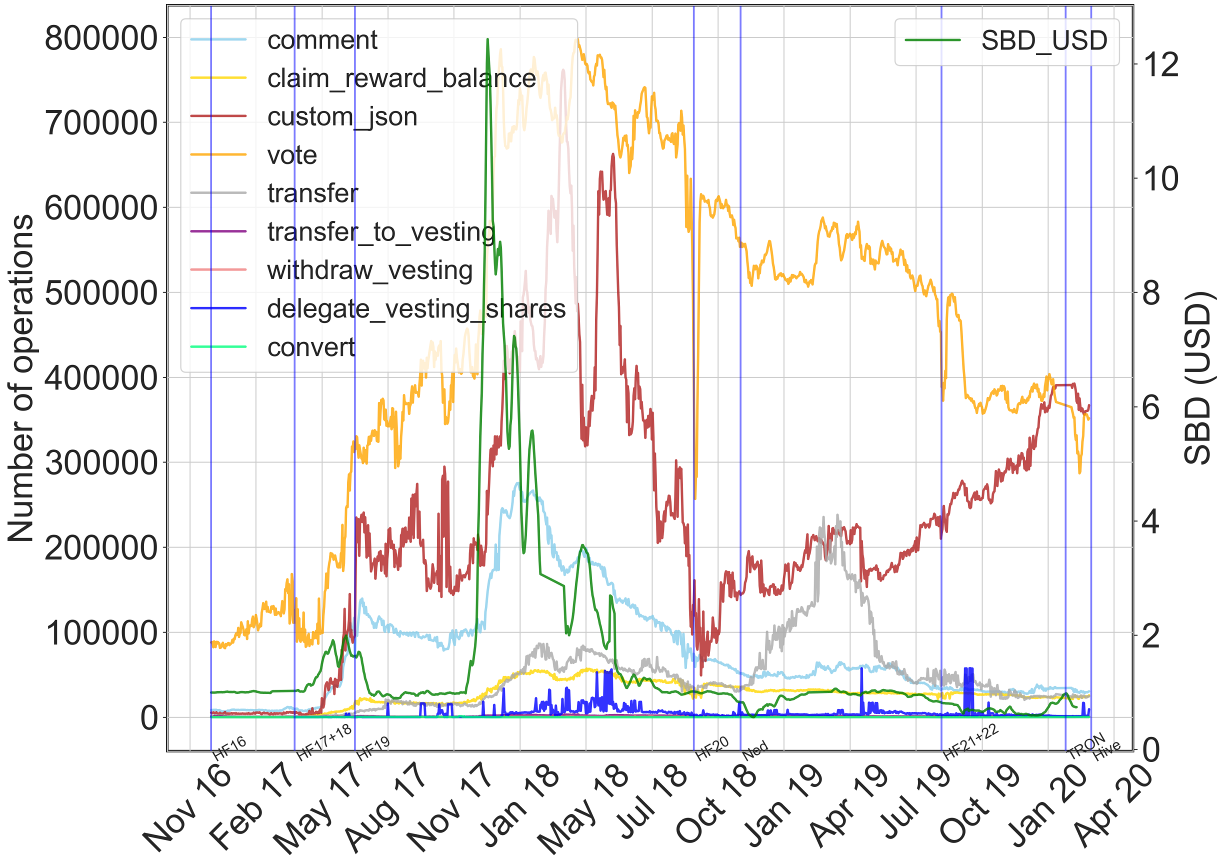

Fig 1. S2 Text. Social/financial action time series and SBD price. Time plots of the daily volume of social and financial operations along with the SBD price in USD (green). On the x-axis: time in days. On the left y-axis: volume of operations per day (visualization of smoothed values, with running average window of 7 days). On the right y-axis: SBD price in USD. The blue vertical lines correspond to important events, like hard forks (HFXX), the crisis announcement by Scott (Ned) - Steemit founder, the selling of the company to TRON Foundation (TRON), and the Hive fork (Hive), which corresponds to the end of the observation period.

Table 1. S2 Text. Social actions and cross-correlations with SBD price. The column "Total" reports the overall volume of operations during the observation period. The second column reports the average daily volume. In the last three columns we report the cross-correlation, the maximum cross-correlation and the lag with the highest cross-correlation, respectively.

Operation	Total	Average	Corr	Max XCorr	Lag (days)
comment	93832667	79654.22	0.58	0.84	35
vote	546677598	464072.66	0.30	0.70	64
custom json (all)	270860412	229932.44	0.23	0.76	100
custom json (followed)	134608190	114268.41	0.37	0.76	46
custom json (unfollowed)	20179192	17130.04	0.38	0.70	46
custom json (muted)	540182	458.56	0.52	0.84	36
custom json (share)	8267940	7018.62	0.52	0.80	19

Table 2. S2 Text. Financial actions and cross-correlations with SBD price. The column "Total" reports the overall volume of operations during the observation period. The second column reports the average daily volume. In the last three columns we report the cross-correlation, the maximum cross-correlation and the lag with the highest cross-correlation, respectively.

operation	Total	Average	Corr	Max XCorr	Lag(days)
claim reward balance	31609874	29708.53	0.27	0.73	41
transfer	55033746	46717.95	-0.05	0.81	457
transfer to vesting	1393465	1182.91	0.36	0.74	42
withdraw vesting	344062	292.07	0.17	0.55	113
delegate vesting shares	5260366	5038.66	0.07	0.32	146
convert	101308	92.77	-0.06	0.46	-167

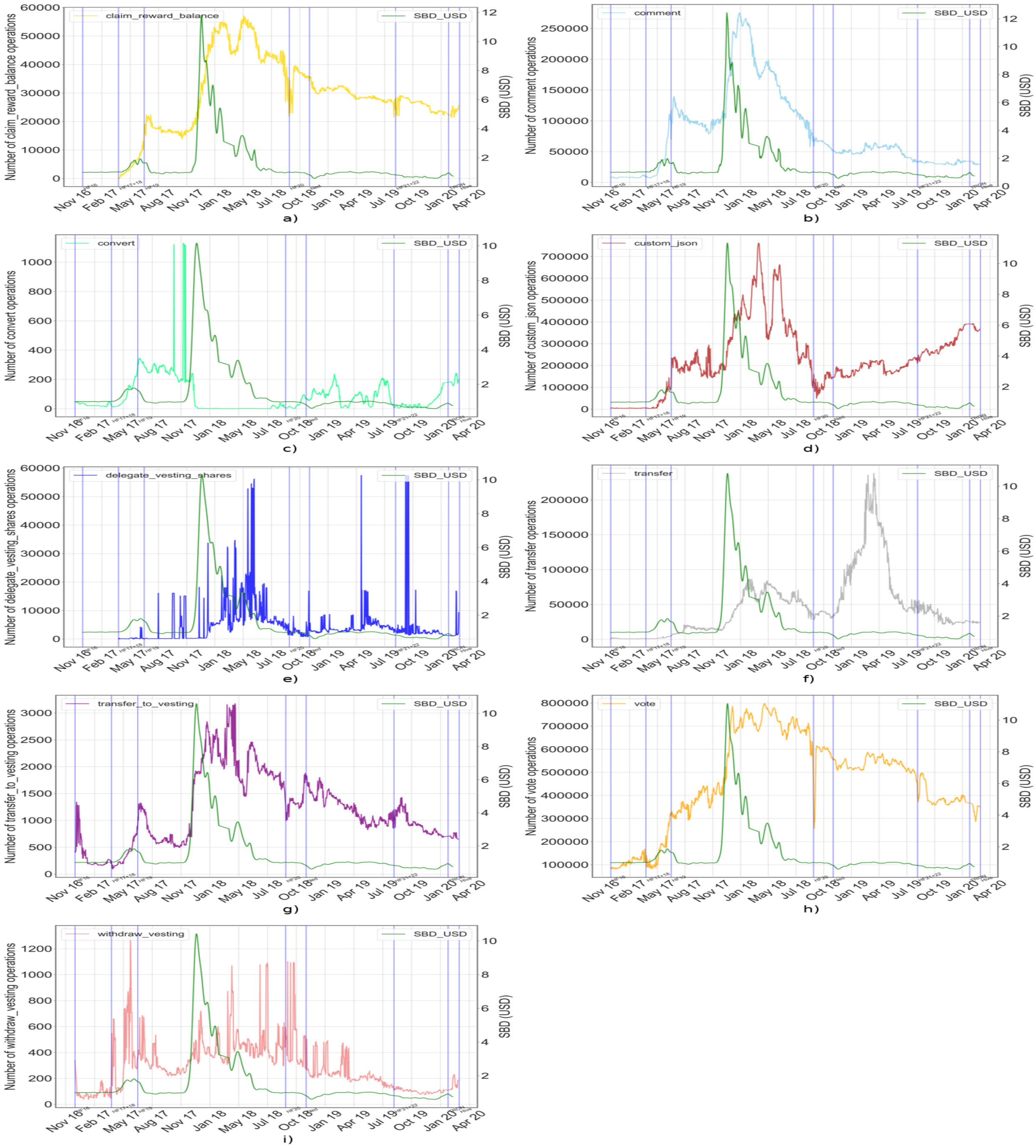

Fig 2. S2 Text. Daily volume of the operations. Time series of the daily volume of the following operations: a) `claim_reward_balance`, b) `comment`, c) `convert`, d) `custom_json`, e) `delegate_vesting_shares`, f) `transfer`, g) `transfer_to_vesting`, h) `vote`, i) `withdraw_vesting`.

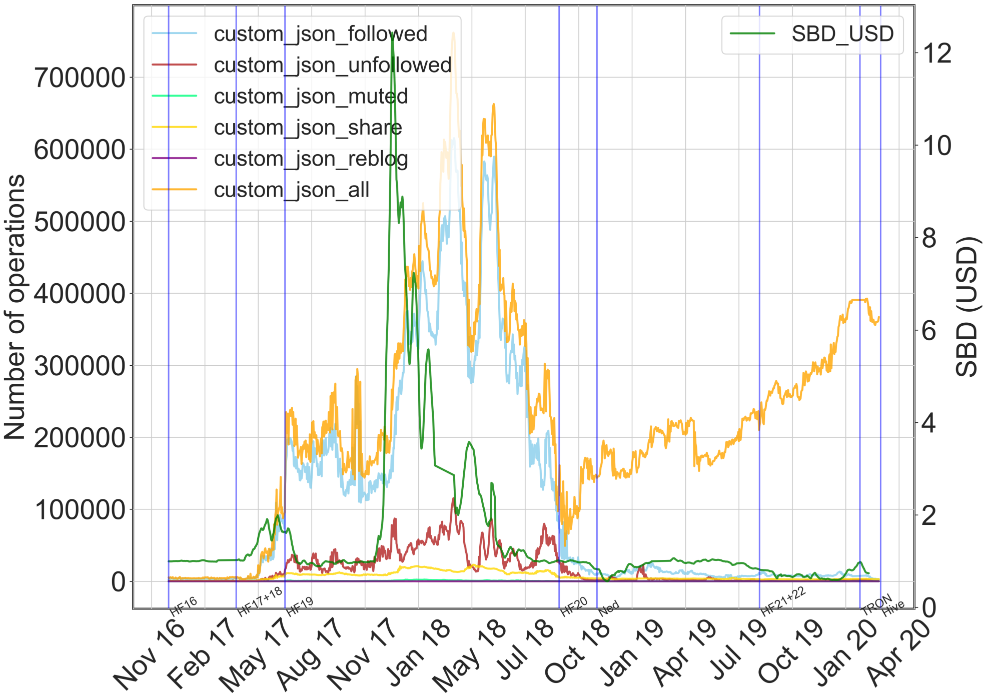

Fig 3. S2 Text. Daily volume of the social actions in `custom_json`. Time series of the main social actions included in the `custom_json` operations: follow, unfollow, mute, share and reblog (retweet): along with the SBD price in USD (green). On the x-axis: time in days. On the left y-axis: volume of actions per day. On the right y-axis: SBD price in USD. The blue lines correspond to important events, like hard forks (HFXX), the crisis announcement by Scott (Ned), the selling of the company to TRON Foundation (TRON), and the Hive fork (Hive).
